# The Energy of Muscle Contraction. I. Tissue Force and Deformation During Fixed-End Contractions

**DOI:** 10.3389/fphys.2020.00813

**Published:** 2020-08-31

**Authors:** James M. Wakeling, Stephanie A. Ross, David S. Ryan, Bart Bolsterlee, Ryan Konno, Sebastián Domínguez, Nilima Nigam

**Affiliations:** ^1^Department of Biomedical Physiology and Kinesiology, Simon Fraser University, Burnaby, BC, Canada; ^2^Department of Mathematics, Simon Fraser University, Burnaby, BC, Canada; ^3^Neuroscience Research Australia, Randwick, NSW, Australia; ^4^University of New South Wales, Graduate School of Biomedical Engineering, Randwick, NSW, Australia; ^5^Department of Physics and Astronomy, University of British Columbia, Vancouver, BC, Canada

**Keywords:** muscle, energy, finite element model, MRI, contraction, tissue, deformation, 3D

## Abstract

During contraction the energy of muscle tissue increases due to energy from the hydrolysis of ATP. This energy is distributed across the tissue as strain-energy potentials in the contractile elements, strain-energy potential from the 3D deformation of the base-material tissue (containing cellular and extracellular matrix effects), energy related to changes in the muscle's nearly incompressible volume and external work done at the muscle surface. Thus, energy is redistributed through the muscle's tissue as it contracts, with only a component of this energy being used to do mechanical work and develop forces in the muscle's longitudinal direction. Understanding how the strain-energy potentials are redistributed through the muscle tissue will help enlighten why the mechanical performance of whole muscle in its longitudinal direction does not match the performance that would be expected from the contractile elements alone. Here we demonstrate these physical effects using a 3D muscle model based on the finite element method. The tissue deformations within contracting muscle are large, and so the mechanics of contraction were explained using the principles of continuum mechanics for large deformations. We present simulations of a contracting medial gastrocnemius muscle, showing tissue deformations that mirror observations from magnetic resonance imaging. This paper tracks the redistribution of strain-energy potentials through the muscle tissue during fixed-end contractions, and shows how fibre shortening, pennation angle, transverse bulging and anisotropy in the stress and strain of the muscle tissue are all related to the interaction between the material properties of the muscle and the action of the contractile elements.

## Introduction

Most of our understanding of muscle function and performance comes from measurements at small scales such as sarcomeres, single fibres and small muscles. Additionally, muscle contraction data have typically been determined when muscle is fully active, changes length at constant velocity, and considers forces and length changes in only the muscle's longitudinal direction. By comparison, we know much less about how whole, large muscles contract, particularly when they are not fully active and contract with varying velocities. Yet, these are exactly the conditions that we may want to understand in order to understand healthy muscle function, and the impairments that arise from injury, disuse and disease. Understanding how the contractile elements interact with the tissue properties of the whole muscle, how deformations may arise in all three dimensions during contraction, and how the dynamics of muscle size influences whole muscle performance may result in muscle behaviours that are not intuitive from the understanding of single fibre function alone. The purpose of this series of papers is to consider contractile mechanisms that are relevant at the whole muscle level, and how these influence the design and performance of skeletal muscle.

Muscles change shape and develop forces when they contract. These effects are typically assumed to occur along the longitudinal direction of the muscle, however, shape changes and forces can occur in all three dimensions. For example, as a muscle shortens then it must increase in girth, or cross-sectional area, in order to maintain its volume (Zuurbier and Huijing, 1993; Böl et al., 2013; Randhawa and Wakeling, 2015). Additionally, as a muscle expands in cross-section it will tend to push outwards as transverse forces develop. Indeed, transverse expansions have been reported from early studies, where contracting muscle bulged to fill glass tubes (Swammerdam, 1758, source: Cobb, 2002), to more recent studies where muscle bulging has been reported in both animal (Brainerd and Azizi, 2005; Azizi et al., 2008) and human studies (Randhawa et al., 2013; Dick and Wakeling, 2017). Transverse forces and deformations have also been recorded by muscles lifting weights when they bulge (Siebert et al., 2012, 2014; Ryan et al., 2019), which is akin to lifting your body by tensing your glutes whilst you are seated.

The 3D shape changes of a muscle are important to its function (Azizi et al., 2008). The forces that muscle fibres actively develop decrease the faster they shorten (Hill, 1938), and thus processes that affect fibre shortening velocity will also affect their force. As the fibres shorten then they must expand in girth to maintain their volume, making the fibres press on each other in a transverse direction. In pennate muscle this transverse expansion is accommodated by the fibres rotating to greater pennation angles (Alexander, 1983; Maganaris et al., 1998), or expanding in either of the two transverse directions (Wakeling and Randhawa, 2014; Randhawa and Wakeling, 2018). The increases in pennation angle result in lower fibre shortening velocity allowing the fibres to develop greater forces, in a process known as muscle belly gearing (Wakeling et al., 2011). The forces developed by whole muscle affect how it changes shape and can cause gearing to vary (Dick and Wakeling, 2017), with this variable gearing favouring velocity output at low loads and force output against high loads (Azizi et al., 2008). Changes to the 3D shape of muscle therefore influence the deformations and speeds at which the fibres shorten, and consequently affect whole muscle forces.

Transverse forces acting at the surface of muscle are also important to muscle function. When groups of muscles within anatomical compartments contract together, their transverse bulging causes the muscles to press on each other, and this results in lower forces being generated by the collective group of muscles than is possible by the sum of the muscle forces if they are isolated (de Brito Fontana et al., 2018). In a similar manner, when compressive forces are applied to the transverse surfaces of contracting muscle, the muscle forces generated along their longitudinal direction decreases (Siebert et al., 2012, 2014, 2016, 2018; Ryan et al., 2019), and the deformations of the fibres, changes in pennation angle and belly gearing are also affected (Wakeling et al., 2013; Ryan et al., 2019). Additionally, the tendency for a muscle to bulge can cause the muscle to experience internal work: generating a transverse force that can lift a weight (Siebert et al., 2012, 2014). Contracting muscle thus develops and reacts to transverse forces acting on its surface, and when the surface deforms in the transverse directions, this will additionally result in work being done.

This paper will consider the effect of work and energy on muscle contractions. Mechanical work is the amount of energy transferred by a force. For clarity in this paper, the term work will be used to describe mechanical work at the surface of the muscle, whereas the term energy will be used to describe the internal energy within the muscle. This internal energy is a strain-energy, which is energy stored by a system undergoing deformation. At the whole muscle level, any process that redistributes energy into a transverse direction will detract from the energy that can be used to generate mechanical work. Currently, we know relatively little about how energy redistributes within the whole muscle structure, and how this redistribution of energy relates to the work done. However, energy redistribution within muscle may have important implications to both the mechanical and metabolic function of a muscle (Williams et al., 2012; Roberts et al., 2019). Surprisingly, these energetic considerations have barely been incorporated into our current understanding of whole muscle function.

This is the first of a series of papers in which we explore how the redistribution of energy within muscle affects its mechanical function during contraction, and we use these energetic mechanisms to demonstrate how whole muscle function is not simply due to the behaviour of individual contractile elements, but rather emerges from the mechanics of the whole 3D muscle structure. In this current paper we present a mechanistic framework for quantifying the energy redistribution and describe how strain-energy is related to the stretch and shortening of the muscle fibres, to the 3D shape and deformations of the whole muscle, and to the forces developed during muscle contraction. We extend this analysis in two companion papers to identify how transverse forces and compression affect the muscle force in its longitudinal direction, and how the mechanical cost of accelerating the inertial mass of the muscle tissue lessens the mechanical performance during dynamic contractions.

## Approach

Experimental and modelling studies have reported local variations in tissue deformations within muscle (Pappas et al., 2002; Higham et al., 2008; Hodson-Tole et al., 2016), and these variations can be explained by the internal mechanics of the muscle fibres and surrounding tissue (Blemker et al., 2005; Rahemi et al., 2014). Thus, how a muscle's tissue deforms during contraction depends on the general structural and material properties of the muscle, rather than on the particular features of the muscle's surface geometry. However, a wide range of muscle sizes, shapes and architectures exist (Wickiewicz et al., 1983; Lieber and Fridén, 2000), so while the same physical principles govern the internal mechanics and behaviour of muscle, the actual tissue deformations and stresses that develop during contraction also depend on muscle shape and architecture (Gans and Bock, 1965; Lieber and Fridén, 2000). Hence, in this study we compare deformations of muscle tissue for geometries that span a range of pennation angles and cross-sectional areas to distinguish general principles that do not rely on specific features of muscle shape.

The premise in this study is that strain-energy redistributes through the muscle tissue resulting in changes to the force and external work of whole muscle. The maximum work from an active sarcomere is given by the area under its force-length curve when it shortens very slowly along its entire range of motion so that its contractile forces are close to their maximum isometric value at each instant (Weis-Fogh and Alexander, 1977), giving a maximum strain energy-density of ~1.5 × 10^5^ J m^−3^, where the strain-energy density is the strain-energy for a given volume of muscle tissue. The maximum muscle work possible would be approximately equal to the product of the work from each sarcomere and the number of sarcomeres in the muscle, or equivalently the product of the strain energy-density from the sarcomere and the volume of the muscle tissue. Here we additionally compare blocks of muscle with different shapes and architectures, but the same initial volumes so that we can evaluate how strain-energy is redistributed within them independently from the effect of muscle size, or effectively the number of sarcomeres.

The mechanics of whole muscle contraction depend on many factors such as the geometry of the muscle and properties of the tissue, and different models have been evaluated to explain how individual factors influence contractile performance. Here we present a general modelling approach, using the principle of minimum total energy (Liu and Quek, 2014), to explain many of these different effects in one framework. Previously, muscle shape changes have been related to belly gearing and shortening velocities using both 2D and 3D geometrical models (e.g., Maganaris et al., 1998; Azizi et al., 2008; Randhawa and Wakeling, 2015); transmission of forces and deformations between the longitudinal direction and transverse directions have been investigated with studies that used fluid and hydrostatic models and experiments (Azizi et al., 2017; Sleboda and Roberts, 2017); and lumped parameter models have accounted for tissue mass, accelerations and the mechanical cost of inactive tissue (Günther et al., 2012; Ross and Wakeling, 2016; Ross et al., 2018b). However, the physical principles that relate muscle shape and force should emerge from the complex interactions between the contractile elements, the material properties of the tissue and the 3D structure of the muscle and not rely on specific explanations for distinct examples. Modelling muscle as a fibre-reinforced composite biomaterial allows the principles of continuum mechanics and the finite element method (FEM) to be applied to this problem (Johansson et al., 2000; Meier and Blickhan, 2000; Yucesoy et al., 2002; Oomens et al., 2003; Blemker et al., 2005; Röhrle and Pullan, 2007; Böl and Reese, 2008; Rahemi et al., 2014): in this approach tissue deformations are associated with an energy function, usually called the strain-energy function. The strain-energy function describes all the active, passive and incompressibility behaviours of the muscle tissue, allowing these models to track the redistribution of strain-energy potentials within the tissue. Thus, such FEM models are ideal for evaluating the redistribution of energy within a contracting muscle.

In this paper we use a FEM model of muscle that we previously developed (Rahemi et al., 2014, 2015; Ross et al., 2018b), but with a number of numerical and computational refinements. Both muscle and aponeurosis tissue are modelled as fibre-reinforced composite biomaterials using the principles of continuum mechanics. For the muscle tissue, the fibres in the model represent the myofilaments that develop both active and passive forces (from the actomyosin cross-bridges and titin molecules, respectively). These model fibres are non-linear actuators, with their forces being calculated using a Hill-type modelling approach (e.g., Zajac, 1989). The fibres develop active forces that increase with the activation level and their orientations are specified at each point, allowing the pennation angle to be calculated. The material properties of the muscle are modelled as base material (combining both intracellular and extracellular effects), and the whole muscle tissue is considered as nearly-incompressible. The aponeurosis tissue is also fibre-reinforced, but here the model fibres represent collagen fibres within the aponeurosis. Similar to the muscle, the aponeurosis has its own base material properties and nearly incompressible constraints. Both the muscle and aponeurosis tissues are thus transversely isotropic. In this paper we quantify the energy state of the different elements within the model (the contractile strain-energy potential from the fibres, the base material strain-energy potential, and the volumetric strain-energy potential that penalizes volume changes at each element), and track the redistribution of energy between these elements as the fixed-end contractions progress. Here we evaluate the deformations of the medial gastrocnemius between our modelled and magnetic resonance imaging (MRI) results, and we quantify the redistribution of energy that occurred within a block of muscle in the medial gastrocnemius, and across a series of additional blocks with varying geometry and architecture.

## Methods

In this paper we present a parallel modelling and experimental study to evaluate the changes in internal energy during fixed-end muscle contraction. We model blocks of muscle with different sizes, shapes and pennation angles to determine how these features affect the strain-energy, deformations and forces of the muscles. To assess how valid these modelled effects are to whole muscle contractions we compare the model outputs to the outputs of a block of muscle within the experimentally-measured geometry of the medial gastrocnemius muscle. Additionally, we validate the deformations of the medial gastrocnemius that are predicted by the model with experimentally measured deformations of the muscle surface geometry and the internal fibre pennation angle.

### Finite Element Model

#### Formulation

We modelled the muscle tissue as a three-dimensional and nearly incompressible fibre-reinforced composite material. While the model is transversely isotropic, the presence of fibres through the material results in an overall anisotropic response of the tissue. The formulation of our model is based on the balance of strain-energy potentials proposed by Simo et al. (1985); see also Simo and Taylor (1991), Weiss et al. (1996), and Blemker et al. (2005). Our approach is to numerically approximate the displacements **u**, internal pressures *p*, and dilations *J* of the tissues so that the total strain-energy of the system *E*_tot_ reaches a local optimum. The total strain-energy of the system is given by:

(1) $$\begin{array}{l} E_{\text{tot}}\left(\text{u},\;p,\;J\right)\;=U_{\text{int}}\left(\text{u},\;p,\;J\right)-W_{\text{ext}}\left(\text{u}\right), \end{array}$$

where *U*_int_ denotes the internal strain-energy potential of the muscle and *W*_ext_ denotes the external work on the system. In other words, we seek a state (**u**, *p, J*) such that the first variation of the total strain-energy *DE*_tot_ is zero:

(2) $$\begin{array}{l} DE_{\text{tot}}\left(\text{u},\;p,\;J\right)\;=0. \end{array}$$

To approximate the solutions (**u**, *p, J*) of Equation 2 we used the finite element method, and to approximate the integrals that are computed as a part of this method, we used the quadrature rule which involves quadrature points and weights. Therefore, **u**, *p*, and *J* are only known at the quadrature points. See section Appendix I. Details of Model Formulation for more details on the formulation of our problem. The model was implemented in the finite element library deal.II version 8.5 (Arndt et al., 2017).

#### Material Properties

**Figure 1:**
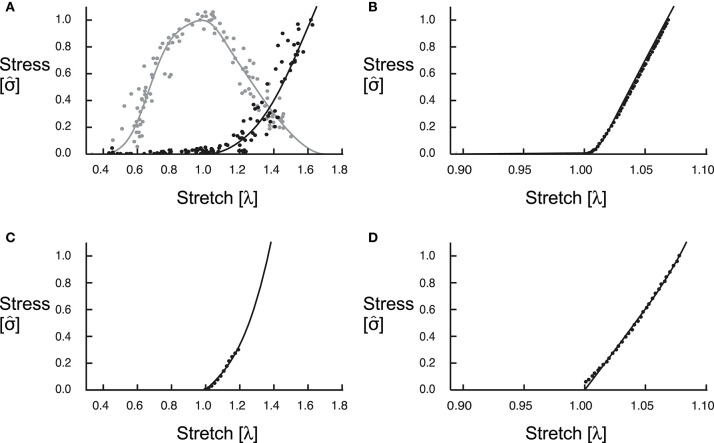
Constitutive relations. We fit curves for the active (grey) and passive (black) along-fibre properties of the muscle tissue to experimental data from Winters et al. (2011) **(A)**. The along-fibre properties of the aponeurosis were due to only passive forces, and we fit this curve to experimental data from Dick et al. (2016) **(B)**. **(C,D)** Show curves for the isotropic base material for muscle, fit to data from Mohammadkhah et al. (2016), and aponeurosis, fit to data from Azizi et al. (2009), respectively.

The fibres in the fibre-reinforced composite material represent the behaviour of the myofilaments in muscle that develop both active (contractile element) and passive (parallel elastic) forces, and the tissue surrounding the fibres that we refer to as base material, represents the behaviour of the additional intra- and extracellular components that include connective tissue such as ECM, blood, and other materials within whole muscle. We formulated the active and passive fibre curves as trigonometric polynomial and second-order piecewise polynomial fits of experimental data (Winters et al., 2011). These curves (Figure 1A) are similar in shape to the Bézier curves presented in (Ross et al., 2018a) but are not parametric. To model the base material properties of the muscle, we used a Yeoh model (Yeoh, 1993) fit to experimental data for tensile loading of muscle in the across-fibre direction (Mohammadkhah et al., 2016) (Figure 1C). Because the properties of the fibres only act in the along-fibre direction, the tensile across-fibre data from Mohammadkhah et al. (2016) likely only represents the properties of the base material surrounding the fibres. We assumed that the base material is isotropic and so contributes to the muscle tissue response in all directions.

While the block geometries in this paper are composed of only muscle and do not account for the effects of aponeuroses, we included both a superficial and deep aponeurosis in the MRI-derived geometries to better replicate the behaviour of the *in vivo* medial gastrocnemius. As with the muscle tissue, we modelled the aponeurosis tissue as fibre-reinforced composite material. However, while the fibres in the muscle tissue produce both active and passive forces, the fibres in the aponeurosis tissue produce only passive forces and represent the behaviour of the bundles of collagen fibres within the connective tissue. Given that tendon is an extension of aponeurosis and likely has similar composition and collagen properties, we fit the passive fibre curve to experimental stress-stretch data for tendon (Dick et al., 2016). This passive fibre curve is of the same form as the piecewise polynomial that we used for the muscle fibre passive curve and can be seen in Figure 1B. To model the base material properties of the aponeurosis, we fit the model from Yeoh (1993) to transverse tensile loading data for aponeurosis (Azizi et al., 2009; Figure 1D).

We modelled both the muscle and aponeurosis tissue as nearly incompressible, with a volumetric strain-energy potential describing the energetic cost of the compression that does occur in the muscle. These volumetric strain-energy potentials were described by their bulk modulus κ, that took values of κ =10^6^ Pa for the muscle and κ =10^8^ Pa for the aponeurosis (Rahemi et al., 2014, 2015; Ross et al., 2018b). Finally, we set the maximum isometric stress of the tissues to 200 kPa.

### Experimental Data Collection

We collected surface geometry and internal architecture data for the medial gastrocnemius (MG) muscle using magnetic resonance (MRI) and diffusion tensor (DTI) images of the lower limb. These data were to provide initial geometries for model simulations of muscle contraction (from the resting condition), and to provide deformed geometries and architectures during fixed-end contraction to validate the simulation outputs from the finite element model of muscle contractions.

Four female participants (age 29 ± 4 years mean ± S.D.) with no recent history of musculoskeletal disease or injury took part in this study. All procedures conformed to the Declaration of Helsinki (2008) and were approved by University of New South Wales' Human Research Ethics Committee HREC (approval HC17106). We obtained written informed consent from all participants. Details of the MRI acquisition and data analysis can be found in Appendix II. Experimental Measurements From MRI and DTI. Briefly, we had participants lie supine in an MRI scanner with their right knee slightly flexed, their right foot strapped to a footplate and their ankle at 5° plantarflexion. We instructed participants to generate plantarflexion torques of 10% (twice) and 20% (once) of their maximum voluntary plantarflexion torque while we imaged their right lower leg: we provided visual feedback of the plantarflexion force to help participants maintain constant plantarflexion torque during the 2.5 min scans.

**Figure 2:**
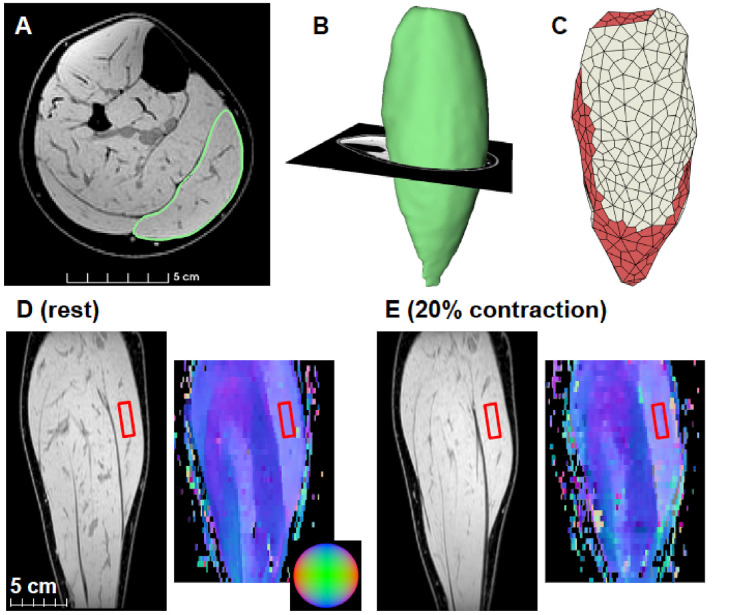
Example MRI scan of medial gastrocnemius and model geometry. **(A)** Example of an axial slice of the mDixon MRI scan of the calf approximately midway between the ankle and the knee. The medial gastrocnemius is outlined in green. **(B)** Example of a three-dimensional surface model reconstruction of the medial gastrocnemius. **(C)** Example of a hexahedral mesh of the same muscles with the muscle elements in red and the aponeurosis elements in white. **(D)** Example of a coronal slice of the mDixon scan (left) and DTI-derived primary eigenvectors shown as a red-green-blue color maps (right) intersecting the medial gastrocnemius mid-belly. These images were obtained with muscle at rest. The sphere in the inset can be used for interpretation of the directions of the primary eigenvectors (blue: superior/inferior, red: left/right, green: anterior/posterior). **(E)** mDixon and primary eigenvector map obtained at the same location in the muscle during a contraction at 20% plantarflexion torque. The red rectangles in the images indicate the 30 × 10 × 10 mm region of the muscle that was used to compare to simulations of a muscle block of similar size.

We calculated fibre orientations from DTI scans (primary eigenvector of the diffusion tensor) and created 3D surface models of the medial gastrocnemius from anatomical MRI scans both while the muscle was relaxed and during contractions (Figure 2).

### Model Simulations

#### Simulations of Block Geometries

**Figure 3:**
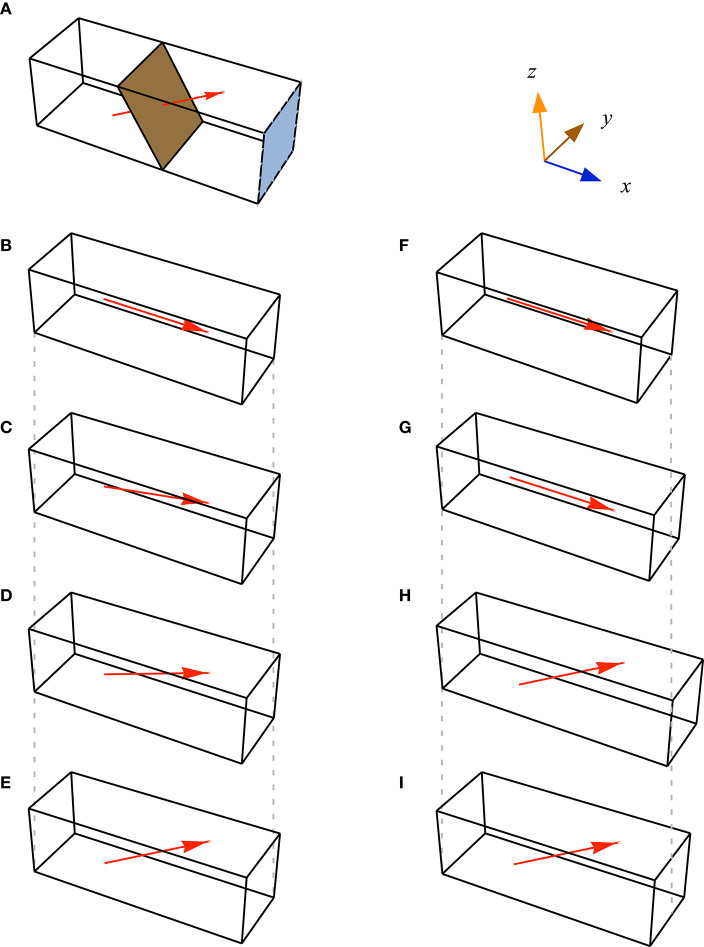
Geometries of muscle blocks. **(A)**. Each block of muscle was defined by its initial cross-sectional area (CSA, blue face, parallel to the *yz* plane): the physiological cross-sectional area (brown plane, normal to the fibre direction) was greater than the CSA for pennate blocks. We set fibre orientations at each quadrature point (red vector, shown here only through centre of muscle). Modelled muscle had orientations defined at 128,000 quadrature points within each block. **(B–E)** Some blocks had the same dimensions, but different fibre orientations. **(F–I)** Other blocks also varied in their CSA and volume. Vertical grey dash lines are projected down from the diagonal corners of blocks **(A,F)** to highlight where the other blocks have changed dimensions.

We constructed a series of 25 blocks of parallel-fibred and unipennate muscle with cuboid geometries and no aponeurosis (Figure 3). We defined the length of the blocks as the distance between the positive and negative *x*-faces in the *x*-direction. The muscle fibres were parallel to each other and the *xz* plane, but oriented at an initial pennation angle β_0_ away from the *x*-direction. We determined the cross-sectional area CSA of each muscle block from its initial configuration *V*_0_ as the area of the cross-section in the *yz* plane. The muscle blocks had faces in the positive and negative *x, y*, and *z* sides (for *V*_0_) that deformed during contraction. One purpose of this study was to identify if the strain-energy is redistributed within the muscle independently from the effect of muscle size or shape. Hence, we varied the shape and pennation angle of the muscle blocks to span a range of architectures. The standard dimensions for the muscle blocks were 30 × 10 × 10 mm, however, we varied CSA and the block volumes *Vol* by 15%, and β_0_ from 0 to 37° Some blocks had the same CSA and *Vol*, but varied in their β_0_; some blocks had the same β_0_ and *Vol* but differed in their CSA; some blocks had the same CSA and β_0_ but differed in their *Vol*. In this manner, the effects of β_0_, CSA, and *Vol* could be independently tested.

We simulated contractions of the muscle blocks using the FEM model. To fix the ends of the muscle blocks, we imposed kinematic constraints on the positive and negative *x* end faces in all three directions. We set the initial length of the fibres to their optimal length (λ_iso_ = 1) and linearly ramped the activation from 0 to 100% over 10 time steps. For these blocks containing only muscle tissue, the simulations would only converge to an activation of 100% when β_0_ was >5°, so we increased the stiffness of the base material using a scaling factor *s*_base_ of 1.5 to allow the model to converge to maximum activation when β_0_ was 5° or less.

#### Simulations of MRI-Derived Geometries

We created hexahedral meshes of the MG muscle at rest for all four participants. To do this, we outlined the shape of the muscle on all scan slices where the muscle was visible and then used these outlines to create a surface model of the muscle with 100 nodes. We converted the surface model to a volumetric tetrahedral mesh and then to a hexahedral mesh in GMSH format using GIBBON Toolbox and custom-built Matlab algorithms (MATLAB, 2018; GIBBON Toolbox: Moerman, 2018).

Large parts of the MG surface are covered by aponeuroses, so unlike the block simulations, we included superficial and deep aponeuroses in these simulations to better mimic the behaviour of the whole muscle during contraction. Aponeuroses are thin and difficult to discern on MRI scans so we identified them as regions where the muscle fibres intersect with the muscle surface: these fibres (4,039–7,745 per participant) were tracked using tractography methods on the DTI data, described in Bolsterlee et al. (2019). We added new hexahedral elements to the outside of the muscle surface where the aponeuroses had been identified. These elements tapered linearly in thickness along the muscle's length so that they were 2 mm thick where they merged with the external tendon and 1 mm thick at the other end (Figure 2). Aponeurosis elements were assigned aponeurosis properties.

The DTI-derived muscle fibre orientations provided an opportunity to populate the MG-based geometry with the actual β_0_ at each point (Chen et al., 2016; Alipour et al., 2017). To achieve this, we sampled local fibre orientations at 2 mm intervals along muscle fibres from DTI-derived fibre tracts, and then assigned fibre orientations to quadrature points of all muscle elements using nearest neighbor interpolation (or extrapolation for the most proximal part of the muscle for which no DTI data were available). We set the fibre orientations of quadrature points inside the aponeurosis to be tangential to the muscle surface with a zero *y*-component, i.e., parallel to the muscle's surface and nearly parallel to the muscle's long axis. We additionally evaluated simulations of the MG-based muscle using constant pennation angle β_0_ through the muscle, to compare directly with results from the isolated muscle blocks.

We simulated fixed-end contractions of the MRI-derived geometries for the medial gastrocnemius, up to 100% muscle activation in increments of 10%, by applying kinematic constraints in all three orthogonal directions to the most proximal faces of the superficial aponeurosis and the most distal faces of the deep aponeurosis.

### Post-Processing and Data Analysis

The model geometries used for the FEM simulations were each bounded by their surface. For the block simulations, we characterised the faces of the blocks (–*x*, +*x*, –*y*, +*y*, –*z* and +*z* faces) for the undeformed state *V*_0_, and then followed these for each deformed state *V*. The length of the muscle block *l* is the distance between the -*x* and +*x* faces and was normalized $\hat{l}$ to the length in the undeformed state. The strain ε between the faces is the change in distance between opposite faces, normalized to their initial separation in the undeformed state. The geometries from the medial gastrocnemius muscles from the MRI scans had no distinct faces and so we characterized the changes in width and depth from the whole surface. We sampled cross-sections of the surfaces at 10% intervals along the muscle length: the width was the maximum width of the section, and the depth was given by the cross-sectional area of that section divided by its width.

We defined muscle bulging as displacement of the muscle's surface in the direction perpendicular to the surface. We calculated bulging using distance maps. A distance map is a 3D regular grid of points in which the absolute value of each grid point equals the distance to the nearest point on the surface model. To determine muscle bulging during contraction, a distance map of the muscle surface at rest *V*_0_ was created. We aligned the surface for the current state *V* with that for the undeformed state *V*_0_ using principal component analysis. The distance map of the muscle at rest was then interpolated at the nodes of the aligned current state. The value associated with each node thus approximates the distance to the nearest point of the muscle at rest, allowing for quantification of muscle bulging patterns: the sign indicates whether a point is inside (negative) or outside (positive) the muscle surface. Muscle bulging was calculated using this same approach for both the MRI geometries, and the FEM simulations of the medial gastrocnemius.

The FEM model calculates tissue properties across a set of quadrature points within each model: 128,000 quadrature points for the muscle blocks, and ~37,000 for the medial gastrocnemius geometries. We defined an orientation and stretch (normalized length) at each quadrature point. The pennation angle in the undeformed β_0_ and current β states were calculated as the angle between the fibre orientations and the *x*-axis: this is an angle in 3D space, similar to the 3D pennation angles defined by Rana et al. (2013). The fibre stretch λ_tot_ gives the normalized length of the tissue in the direction of the fibres at each quadrature points. These pennation angles β and fibre stretches λ_tot_ are thus calculated for local regions within the muscle tissue, and so we sometimes reported them as their mean value across the whole tissue or block.

We calculated forces *F* as the magnitude of force perpendicular to a face or plane within the muscle, and the stress σ as that force divided by the area of that face or plane in the current state of the simulation.

The strain-energies are initially calculated as strain energy-densities ψ, which are the strain-energy for a given volume of tissue, in units J m^−3^. The FEM calculates ψ for every quadrature point, and so we calculated the overall strain energy-density from the weighted mean of ψ, where it is weighted by the local dilation at each quadrature point. The strain-energy potential *U* is the strain-energy in the tissue, in units of Joules. We calculated *U* by integrating ψ across the volume of muscle tissue.

We compared the simulation results for isolated blocks to the results of the MRI-based model of the medial gastrocnemius. Specifically, quadrature points inside a 30 × 10 × 10 mm region in the centre of the medial gastrocnemius (Figure 2) were compared to the results for an isolated block of muscle tissue of the same size. For both blocks we used β_0_ = 25°, and *s*_base_ = 1.

Symbols used to reference the post-processing parameters are shown in Table 1.

**Table 1:** Symbols and definitions of variables in the main text.

**Symbol**	**Definition**
**u**	displacement vector
*p*	internal pressure
*J*	dilation
*E*_tot_	total strain energy
*U*	strain-energy potential
*U*_int_	internal strain-energy potential
*W*_ext_	work done by external forces
*DE*_tot_	first variation of *E*_tot_
ψ	strain energy-density
*V*_0_	initial configuration
*V*	current configuration
CSA	cross-sectional area
β_0_	pennation angle in initial configuration *V*_0_
β	pennation angle in current configuration *V*
$\bar{\beta }$	Mean pennation angle in current configuration *V*
*Vol*	current volume
λ_iso_	isovolumetric stretch
${\bar{\lambda }}_{\text{tot}}$	mean total stretch
*s*_base_	stiffness parameter for muscle base material
ε	scalar strain
*l*	length
$\hat{l}$	normalized length
*F*	force
*F*_x_	force in *x*-direction
σ	scalar stress
κ	bulk modulus

## Results

### Simulations of Block Geometries

**Figure 4:**
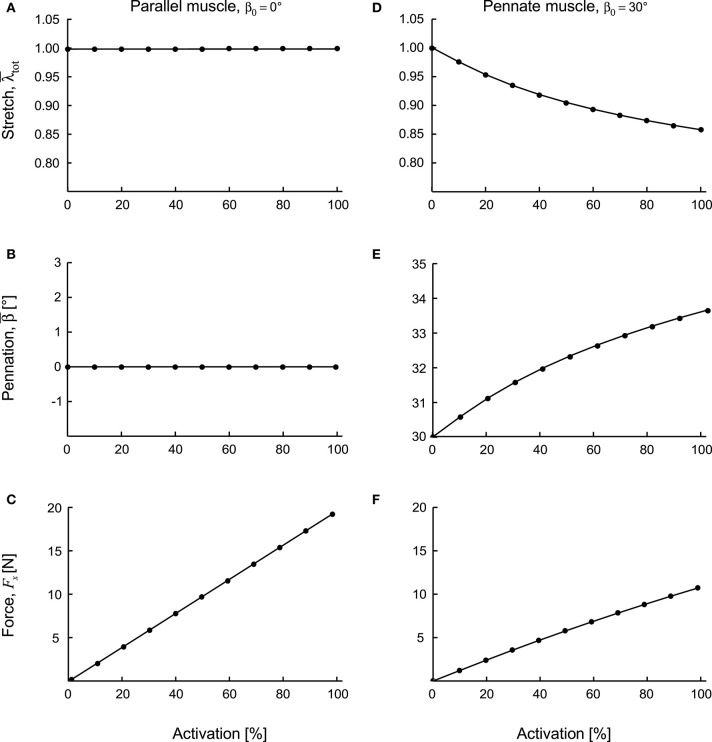
Contractile features for parallel and pennate blocks of muscle during fixed-end contraction. Parallel fibred muscle **(A–C)** and pennate fibred muscle blocks **(D–F)** shown as activation level increased to 100%. The stretch **(A,D)** and pennation angles **(B,E)** are shown as means calculated across the 128,000 quadrature points in each muscle block. The force *F*_*x*_ is in the line or action of the muscle acting on the *x*-face of the block **(C,F)**. These two muscle blocks had the same volume and the same cross-sectional area of 1 × 10^−4^ m^2^.

The parallel fibred (β_0_ = 0°) blocks had their initial fibre direction parallel to the longitudinal direction of the muscle blocks (*x*-direction), and so their fibres showed no net shortening for these fixed-end contractions. Instead, the volume of the blocks showed a marginal increase during contraction, with the fibre stretch ${\bar{\lambda }}_{\text{tot}}$ increasing minimally (Figure 4A). The mean pennation angle for the parallel fibred block was $\bar{\beta }=0$ at full activation. On the other hand, the fixed-end constraints were not in the same direction as the fibre orientation for the pennate (β_0_ > 0°) blocks, and so their fibres underwent a net shortening during contraction (Figure 4D). Indeed, at 100% activation the β_0_ = 30° block shortened to ${\bar{\lambda }}_{\text{tot}}=0.86$, and its pennation angle increased to 33.6° (Figures 4D,E).

**Figure 5:**
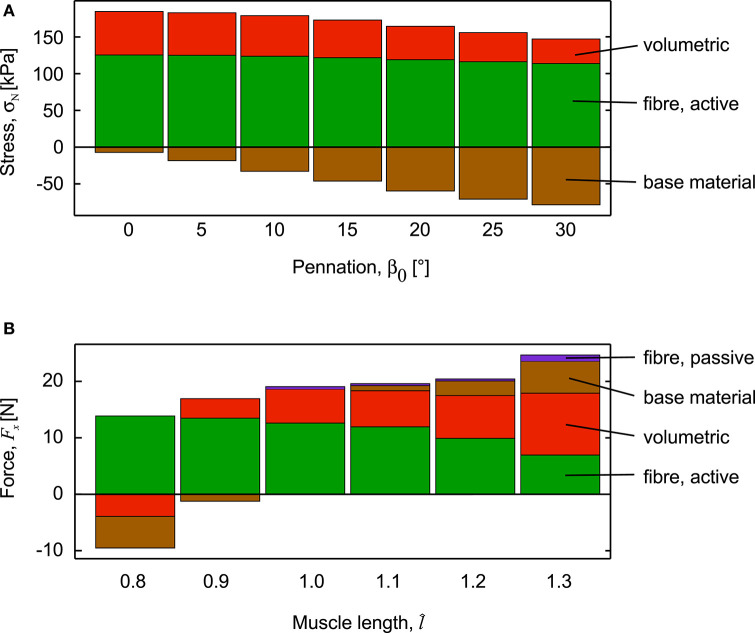
Components of stress and force during fixed-end muscle contraction. **(A)** Stress through the centre of the muscle blocks, normal to the mean fibre direction. Stress is shown for the 100% activation condition, for muscle blocks with initial length λ_iso_ =1, but different initial pennation angle, β_0_. The total stress, normal to the fibres has components from the volumetric, active-fibre and base material strain-energy potentials. Stress is positive if it acts to shorten the fibres, but negative if it acts to length the fibres: the volumetric stress acts to shorten the fibres, but this is resisted by the base material that acts to lengthen the fibres. **(B)** Force in the longitudinal direction of the muscle blocks, measured on the *x*-face. The muscle block had initial pennation β_0_ = 0°, was stretched or shortened to a new length using traction on the +*x* face, then held it at that length as we increased activation to 100%. Note how the base material and volumetric forces oppose shortening at short lengths, and how the passive forces have been redistributed across passive-fibre, base material and volumetric components for longer lengths.

Stresses normal to the mean fibre direction, through the centre of the muscle blocks, increased as activation increased and are shown for the fully active conditions (Figure 5A). These stresses had components due to the different strain-energy potentials. The stresses due to the active-fibre and the volumetric strain-energy potentials both acted to shorten the fibres, whilst the stress from the base material acted to resist shortening. For the parallel-fibred case (β_0_ = 0°), the stress from the volumetric component was a large proportion of the total stress, and there was little resisting stress from the base material. These features transitioned as the pennation angle increased, and the β_0_ = 30° block had the least stress from the active fibre strain-energy potential, and the greatest resistive stress from the base material strain-energy potential. As the pennation angle increased, the normal stress to the fibres had a smaller component in the longitudinal direction (*x*-direction) of the blocks. Indeed, the parallel fibred block (β_0_ = 0°) developed a force of *F*_*x*_ = 19.06 N in its longitudinal direction, whereas the pennate block (β_0_ = 30°) developed a reduced force of 10.70 N (Figures 4C,F).

**Figure 6:**
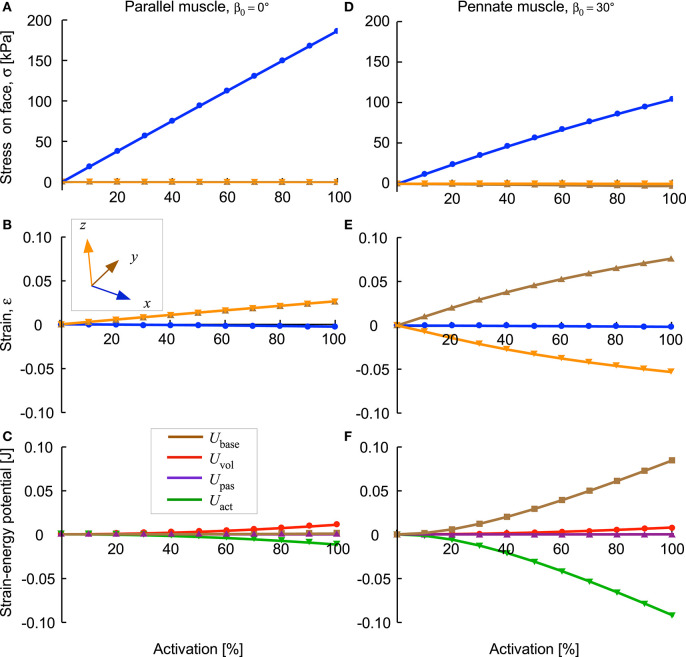
Stress, strain and strain-energy potentials for parallel and pennate blocks of muscle during fixed-end contraction. Parallel fibred muscle **(A–C)** and pennate fibred muscle blocks **(D–F)** shown as activation level increased to 100%. The *y*- and *z*-stresses on the *y*- and *z*- faces were minimal, but were higher within the blocks (see text). The *y*-strain on the *y*-face was the same as the *z*-strain on the *z*-face for the parallel fibred block **(B)**, however, the transverse anisotropy in the stress caused a transverse anisotropy between *y*-strain on the *y*-face and the *z*-strain on the *z*-face for the pennate fibred block **(E)**. The base material strain-energy potential was much larger for the pennate block **(F)** than for the parallel fibred-block **(C)**, and was largely balanced by the active-fibre strain-energy potential. These two muscle blocks had the same initial volume (3 × 10^−6^ m^3^) and same cross-sectional area (1 × 10^−4^ m^2^).

**Figure 7:**
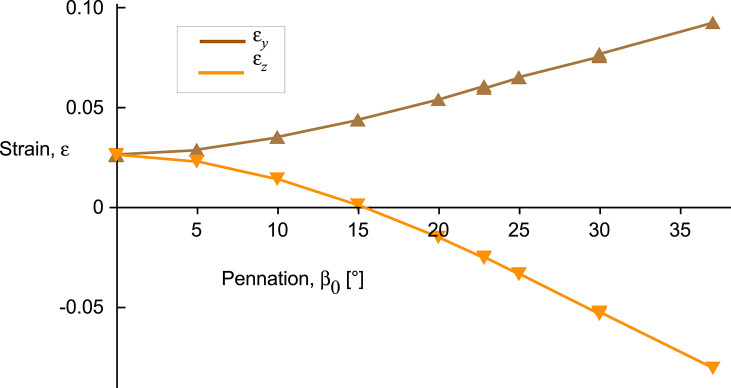
Transverse strains between the *y*- and *z*-faces of the muscle blocks as a function of pennation angle. *y*-strain ε_*y*_ shown for the *y*-face and *z*-strain ε_*z*_ shown for the *z*-face. Results shown for the 100% activation state for all blocks, covering a range of volumes, cross-sectional areas, and pennation angles.

**Figure 8:**
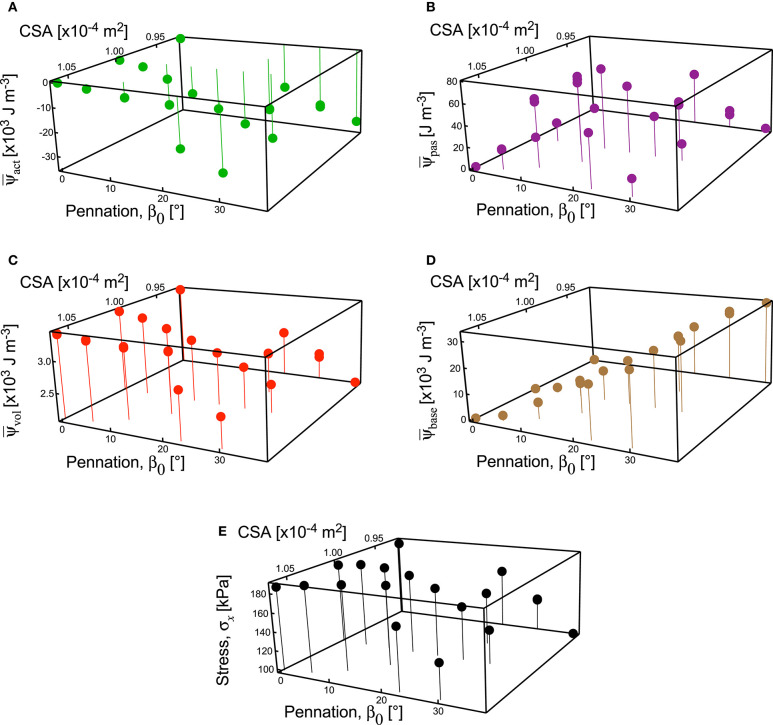
Strain energy-densities and stress in the longitudinal direction for muscle blocks across all geometries. Strain energy-densities ψ are shown for the active-fibre **(A)**, passive-fibre **(B)**, volumetric **(C)**, and base material **(D)** components. Note the different scales for the components of strain energy-density, showing much lower strain energy-density for the passive-fibre and volumetric components. The strain energy-densities presented are relative to the undeformed state *V*_0_. Stress σ_*x*_ in the *x*-direction on the *x*-face **(E)**. Results are shown for the 100% activation state from the simulations.

The *x*-stress on the *x*-face increased as activation increased (Figures 6A,D), but there was no *x*-strain due to these contractions being fixed at their *x*-faces. By contrast, the *y*-stress on the *y*-face and the *z*-stress on the *z*-face were minimal, due to these faces being unconstrained. Nonetheless, stresses in the *y*- and *z*-directions developed within the blocks of muscle when the muscle activated. Within the blocks, stresses in the *y*- and *z*-directions were transversely isotropic for the β_0_ = 0° block, but showed increasing asymmetry as the pennation angle increased. In general, the *y*- stress was larger than the *z*-stress and both acted to expand the muscle block, however, at larger pennation angles (β_0_ > 25°) the *z*-stress became minimal or compressive. The muscle blocks deformed in 3D. For both the parallel and pennate example, the *x*-faces remained fixed, and so no *x*-strain was recorded. For the parallel-fibred block, the small increase in volume resulted in a small, but isotropic, strain in the *y*- and *z*-directions (Figure 6B). There was a transition pennation angle at β_0_ = 15° below which the *z*-strain was positive with the *z*-faces increasing in separation, and above which the *z*-strain was negative with the *z*-faces becoming closer during contraction (Figure 7). Small changes in the active-fibre strain-energy potential in the parallel-fibred block were largely balanced by increases in the volumetric strain-energy potential: here the changes in passive-fibre and base material strain-energy potentials were much smaller (Figure 8C). By contrast, the active-fibre strain-energy potential showed a larger change in the pennate block of muscle that, in this case, was largely balanced by increases in the base material strain-energy potential: here the changes in volumetric and passive-fibre strain-energy potentials were much smaller (Figure 6D).

When the parallel-fibred muscle block (β_0_ = 0°) was stretched or shortened to different lengths before the contraction began, the balance of the strain-energy potentials changed within the muscle. When the muscle block was fully active, the component of the stress due to the active-fibre strain-energy potential acted to shorten the muscle at all muscle lengths tested. The components of stress due to the volumetric and base material strain-energy potentials both acted to resist shortening at the short muscle lengths ($\hat{l}<0.9$), and thus contributed to a reduction to the force in the longitudinal direction *F*_*x*_ (Figure 5B). At the longer muscle lengths, the components of stress due to the volumetric, base material and passive-fibre strain-energy potentials all acted to resist lengthening ($\hat{l}<1.1$). Interestingly, the contribution of the passive-fibre to the overall resistive force was less than that for the base material and also the volumetric components (Figure 5B).

The components of the strain energy-density showed little change with cross-sectional area of the muscle blocks, but a pronounced change with pennation angle (Figure 8). There were very few points in the muscle blocks that showed an increase in fibre stretch at full activation, and so the strain energy-density for the passive-fibre component was small for these simulations (Figure 8B). However, the strain energy-density for the base material increased in an almost linear fashion with pennation angle (*r*^2^ = 0.99: Figure 8D). The strain-energy potential from the base material acted to resist the fibre shortening, and the strain-energy potential from the volumetric and active-fibre components acted to shorten the fibres (Figure 5A). The strain energy-density for the active fibres increased in magnitude at greater pennation angles (Figure 8A), whereas the volumetric component of the strain energy-density decreased at higher pennation angles (Figure 8C). The stress in the longitudinal direction of the muscle blocks (*x*-stress on *x*-face) remained high for pennation angles up to 15–20° (Figure 8E) and showed substantial reduction for pennation angles >20° (Figure 8E).

**Figure 9:**
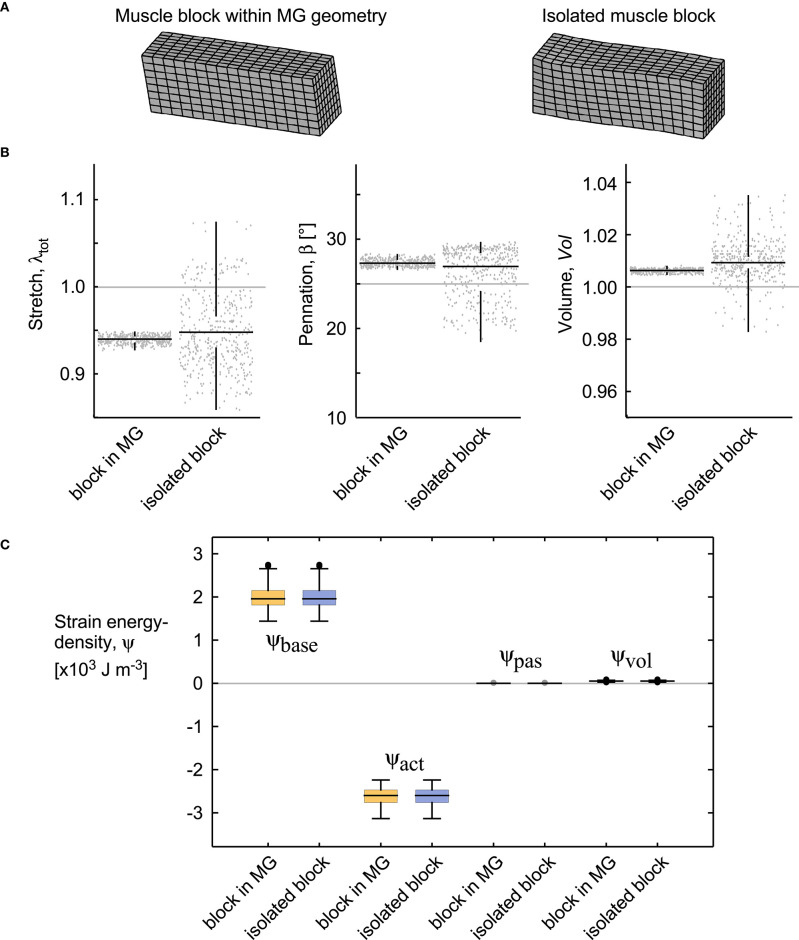
Deformations and energies for a block of muscle within the medial gastrocnemius and an isolated block. Both simulations were evaluated for β_0_ = 25° and 20% activation (similar to the extreme conditions in Figures 10, 12). **(A)** Geometries of the muscle blocks. **(B)** Stretch, pennation angle, and volume shown for each quadrature point, the median for these values (horizontal black bar), and the spread of the data (vertical black bar). **(C)** Strain energy-densities for these blocks. The boxes show the 25 and 75% quantiles, with the median value indicated in the middle. The grey bars show the values for the undeformed state *V*_0_.

The isolated block of muscle showed similar deformations and strain-energy densities as to the block of similar size and fibre orientation extracted from the simulation in the MRI-derived MG geometry (with β_0_ = 25° for both; Figure 9B). There was a greater spread of values in the isolated block, due to the proximity of the fixed-end constraints on the faces, however, the median fibre strain, dilation, and pennation angle were different by <1% or 1° for these simulations. Additionally, the strain energy densities had a close match for the two conditions (Figure 9C).

### Simulations of MRI-Derived Geometries

**Figure 10:**
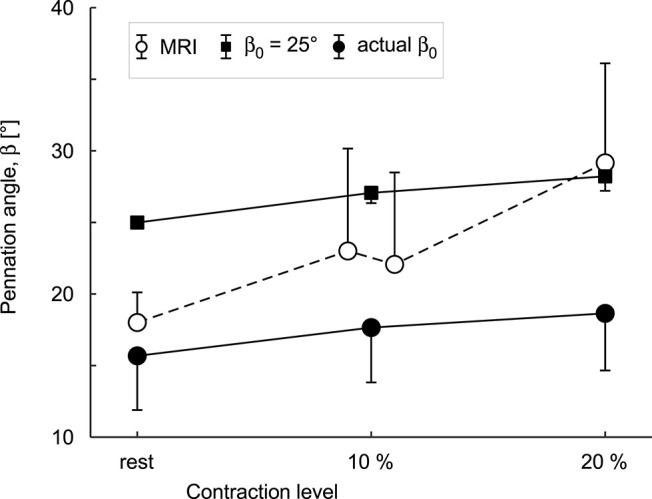
Mean pennation angles ($\bar{\beta }$) in a block of 30 × 10 × 10 mm in the middle of the medial gastrocnemius muscle belly. Data are shown for muscles at rest and during 10% and 20% plantarflexion torques as predicted by the simulations (black squares for $\beta_{0}=25^{∙}$ and black circles for actual β_0_) and as measured from DTI scans (white circles). Circles and error bars are the means and standard deviations of the data/models from four participants. Results are shown for simulations with the DTI-derived initial fibre orientations (actual β_0_) and for simulations in which β_0_ initial was set to 25° so that the simulated fibre orientations at 20% activation were a closer match to the orientations measured with DTI at 20% plantarflexion torque.

**Figure 11:**
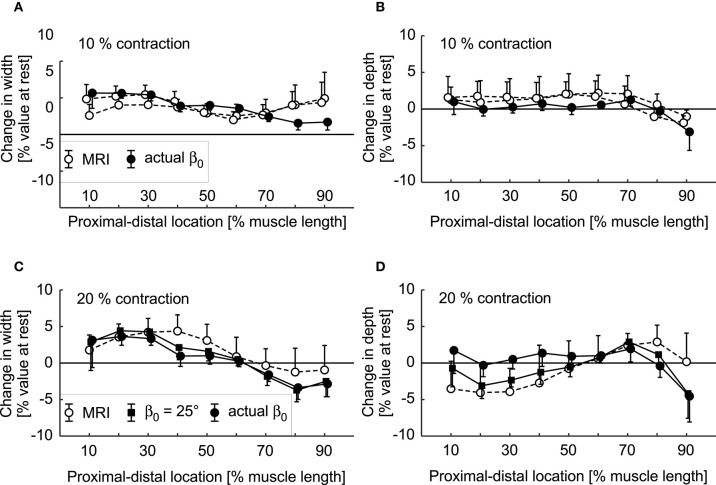
Change in muscle width **(A,C)** and depth **(B,D)** during fixed-end contractions of the medial gastrocnemius. Data are shown for 10% **(A,B)** and 20% plantarflexion torques **(C,D)** as predicted by the simulations (black squares for β_0_ = 25° and black circles for actual β_0_) and as measured from anatomical MRI scans (white circles). Circles and error bars are the means and standard deviations of the data/models from four participants. The two sets of white circles in the top panels are repeated MRI measurements. The bottom panels show changes in depth and width for simulations where the initial fibre orientations (not activated) were derived from DTI (black circles), and where the initial fibre orientations were set to 25° (black squares) so that the simulated fibre orientations at 20% activation were a closer match to the orientations measured with DTI at 20% plantarflexion torque.

**Figure 12:**
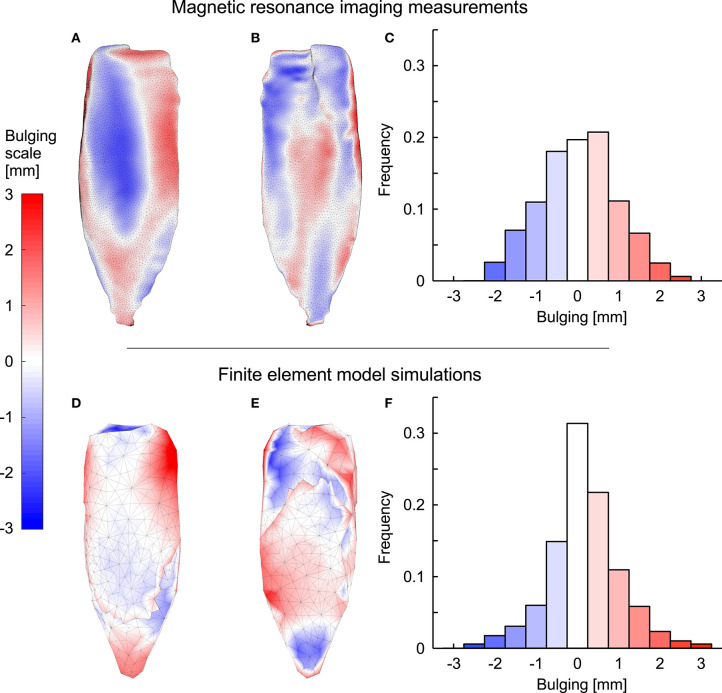
Muscle bulging in the medial gastrocnemius during fixed-end contraction. **(A–C)** show MRI data and **(D–F)** show data predicted by the FEM model for the MRI-derived geometry for one subject where we set the initial muscle fibre orientation β_0_ to 25°. Red and blue shades indicate outwards and inwards bulging [in mm] at 20% activation, respectively. **(A,D)** show the superficial surface of the muscle whereas **(B,E)** show the deep surface. The proportions (frequency) of the points on both surfaces that showed different magnitudes and directions of bulging are shown in **(C,F)**.

The simulations and the DTI data both showed increases in pennation angle β during contractions (Figure 10). However, this increase was larger for the DTI data (11° at 20% plantarflexion torque) than the simulations (3°). The simulations and the MRI data both showed relatively small changes (<2%) in muscle width and depth at 10% activation. At the most distal end, the model decreased its depth slightly whilst the depth increased in the most proximal regions (Figures 11A,B). Changes in width and depth were larger and more heterogeneous along the muscle's length (Figures 11C,D) at 20% activation. The proximal region increased in width and decreased in depth while the distal part decreased in width and increased in depth. On average, changes in width were similar between simulations and MRI measurements. However, the simulation with DTI-derived fibre orientations did not predict the decrease in depth observed in MRI data for 20% activation. Adjusting the initial pennation angle of the model to β_0_= 25° resulted in a closer match between DTI-derived and simulated fibre orientations at 20% activation (Figure 10), and a close match in magnitude and pattern of muscle depth change between MRI and simulations (Figures 11C,D). The adjusted model also resulted in a close match of 3D muscle bulging patterns predicted by the model and as measured from MRI (Figure 12).

## Discussion

This study investigates the energetic mechanisms within muscle tissue during fixed-end contractions. The pennate blocks of muscle (β_0_ > 0°) that we modelled showed general features of contraction that have been typically reported in both animal and human studies (Kawakami et al., 1998; Héroux et al., 2016). The fibres shortened (λ_tot_ < 1) and rotated to greater pennation angles during contraction, even though the ends of the blocks were fixed (Figure 4). An asymmetry developed to the stress in the transverse (*yz*) plane when the muscle was active. Changes to the tissue shape were governed by the isotropic base material properties and the volumetric strain energies: because there was an asymmetry to the stress across the muscle, this resulted in an asymmetry to the transverse tissue deformation that was dependent on pennation angle (Figure 7). In particular, differences in the direction of the *z*-direction strains during contraction were similar to those reported by Chi et al. (2010). These findings explain a mechanism that can result in transverse anisotropy within a muscle, that we have previously reported (Randhawa and Wakeling, 2018).

Interestingly, the volume of the muscle blocks increased during contraction to a small extent (0.6–0.9% for the 20% activation MG simulations; Figure 9B). The mechanism for this increase is described in section Strain-Energy Distribution Through Contracting Muscle. The extent of the increase in volume is related to the choice of the bulk modulus κ of the tissue that is used to calculate the volumetric strain-energy potential. However, previous studies have shown that κ can be varied across a wide range of magnitudes and still result in similar predictions of tissue deformation (Gardiner and Weiss, 2000), and here we used a value consistent with our previous studies (Rahemi et al., 2014, 2015). Our finding that muscle volume can change is consistent with a number of previous studies investigating muscle at different scales (Neering et al., 1991; Smith et al., 2011; Bolsterlee et al., 2017). Intriguingly, the volume of muscle tissue will tend to increase with the muscle bulging transversely, even for a fixed-end contraction of parallel-fibred blocks (zero pennation angle). However, this is consistent with the finding that regions of single fibres can increase in volume during fixed-end contraction (Neering et al., 1991). Local increases in volume had previously been explained due to cytoskeletal effects (Neering et al., 1991); in our simulations the cytoskeleton is represented as part of the base material, and we show that as energy is redistributed to the base material and volumetric components, there is a tendency for the volume to increase. The changes in volume were not uniform through the blocks of muscle. Indeed, variations in bulging along a muscle belly have also been reported in both human and rabbit muscle (Böl et al., 2013; Raiteri et al., 2016). It should be noted that increases in intramuscular pressure during contraction may expel blood from the muscle (Barnes, 1986; Sjøgaard et al., 1988), acting to decrease the whole muscle volume; this may occlude local increases in volume of the muscle tissue due to the volumetric strain-energy potential.

Evaluating the muscle model within the actual MRI-derived geometry of the medial gastrocnemius allowed us to qualitatively validate the outputs from the model. When muscle contracts it develops force and changes length (or more exactly changes shape in 3D). Direct measures of muscle force are virtually impossible to make in humans, and even in the few animal studies where they are measured, the forces would typically only be measured in one-dimension. Thus, complete force and deformation data sets for validating 3D muscle models are sparse for animal studies (Böl et al., 2013), and non-existent for human studies. However, 3D muscle models have previously been validated against deformations of contracting muscle for both animal (Tang et al., 2007) and human (Blemker et al., 2005; Böl et al., 2011) studies. MRI allows 3D deformations of the whole muscle geometry to be measured, allowing for validation of the surface deformations that were generated by the muscle model. It should be noted that the MRI images of the medial gastrocnemius were from the intact leg, and thus subject to external forces and boundary constraints (from surrounding tissues) that were not replicated in the model here. Additionally, the MRI images were for muscle contractions with fixed joint angles, however, due to stretch in the tendons the muscle belly would undergo some shortening (~3 mm during 20% plantarlexion torques as measured from the MRI scans), and thus the model constraints should not be considered as an exact match of the MRI experimental situation. Nonetheless, there was a close match of the deformations of the surface geometry between the MRI and FEM model results (Figure 12). Additionally, a block of muscle identified within the actual MRI-derived geometry showed similar patterns of strain-energy densities as for an isolated block of muscle (Figure 9C). These results give confidence that the mechanisms of contraction identified for the blocks of muscle tissue are realistic.

Novel results from this study are that regions of the muscle are displaced inwards, particularly under the aponeuroses, whereas other regions are displaced outwards, predominantly at the ends and edges of the muscle, and this bulging is apparent in both the MRI images and the FEM model results. Previous ultrasound studies have suggested that tissue deformations and volume changes predicted from imaging the middle region of the muscle belly may not represent deformations along the entire muscle if deformations and volume changes vary along its length (Raiteri et al., 2016; Randhawa and Wakeling, 2018), and these suggestions are now supported by the results from this study.

### Strain-Energy Distribution Through Contracting Muscle

When the muscle contracts it increases in its free energy, with this energy being derived from the hydrolysis of ATP to ADP within the muscle fibres (Woledge et al., 1985; Aidley, 1998). There is only finite free energy available from hydrolysis of ATP within the muscle, governed by the availability of nutrients and ATP, therefore, there is a limit to the work that can be done during a muscle contraction. The mechanical work that can be done by a contracting sarcomere in its line-of-action is an intrinsic property of the sarcomere, is given by the area under the active force-length curve, and has an energy-density of ~1.5 × 10^5^ J m^−3^ (Weis-Fogh and Alexander, 1977). Strain-energy potentials develop in the fibres of our FEM model during contraction: these fibres represent the contractile elements within the myofilaments in muscle. Within the myofilaments, the cross-bridges contain energy when they attach between the actin and myosin as part of the cross-bridge cycle (Williams et al., 2010) like a set of taught springs. This energy is partially redistributed to the thick and thin myofilaments (Williams et al., 2012), with additional energy being released during the power stroke of the cross-bridge cycle. Strain-energy potentials are also redistributed to the titin filaments that are large proteins that span from the M-line to the Z-disc (Gregorio et al., 1999) and likely account for the majority of the passive-fibre strain energy. Base material strain-energy potential can develop in the bulk muscle tissue within the muscle fibres (excluding the myofilament fraction), connective tissue surrounding the muscle fibres such as the extracellular matrix, and in sheets of connective tissue that form the aponeuroses, internal and external tendons. Energy is also used to change the muscle volume. Whilst muscle is often assumed to be incompressible, small changes in volume can occur in fibres (Neering et al., 1991), bundles of fibres called fascicles (Smith et al., 2011) and in the whole muscle (Bolsterlee et al., 2017): these changes in volume result in changes to the volumetric strain-energy potential. Additionally, energy is required for the acceleration of the tissue mass within the muscle to overcome its inertia during rapid movements (Ross et al., 2018b). Finally, energy is transduced to mechanical work at the surface of the muscle where the muscle changes shape and exerts forces on surrounding structures (Siebert et al., 2012, 2014).

Muscle force developed in the longitudinal direction is given by the *x*-component of force on the positive and negative *x*-faces from the blocks of muscle in this study. As the strain-energy potentials within the muscle are redistributed between the different components of energy (volumetric, base material and active- and passive-fibre strain-energies), and because the strain-energy potentials in both the base material and volumetric components are distributed across all three dimensions, the force that can be developed in the longitudinal direction will be less than that could be generated by just the contractile elements alone. This is a fundamental consequence of encasing the model fibres (representing the muscles' contractile elements) within the bulk muscle tissue. In addition, the muscle fibres develop non-uniform stretches throughout the muscle (Figure 9B), and so the muscle is further unable to contract with all its fibres at their optimal length, and thus the whole muscle tissue will always contract at forces less than the theoretical maximum isometric force. Heterogeneities in strain along the muscle fibres are increasingly prominent when considering the impact of surrounding tissue, and have been demonstrated both experimentally (Pamuk et al., 2016; Karakuzu et al., 2017) and predicted through modelling studies (Yucesoy and Huijing, 2012). While the model developed in this current study does not have explicit shear properties between the muscle fibres, the strain experienced by a single fibre will impact neighbouring fibres resulting in similar heterogeneous strains on the adjacent fibres. Therefore, the implicit shear properties in the model will likely cause variations to the passive and base material strain-energy potentials when considering muscle *in vivo*. Even when the stresses and forces are considered relative to the fibre orientation, we find that redistribution of strain-energy potentials through the muscle tissues results in contractile stresses (normal to the fibre direction: Figure 5A) being developed by both volumetric and active-fibre components when the fibre stretch λ_tot_ < 1, and additionally from passive-fibre and base material components at longer muscle ($\hat{l}>1$) and fibre lengths (Figure 5B).

Our computational results suggest that parallel-fibred muscle (β_0_ = 0°) bulges slightly due to its base material properties, even when its ends are fixed and there is no series elasticity such as tendon that could allow the muscle belly to shorten. This again can be explained in terms of the energy redistribution. The free energy in the muscle increases during the contraction process and will be redistributed across fibre, base material and volumetric strain-energy potentials. The energetically favourable state identified in our simulations occurred with a small increase in tissue volume, due to transverse expansion of the fibres (in the *yz*-plane). Thus, muscle bulging should not only be considered to be a consequence of muscle shortening leading to an increase in cross-section to maintain a constant volume (e.g., Azizi et al., 2008, 2017; Siebert et al., 2012), but may also occur due to the biological tissues showing small changes in volume, even for fixed-end contractions. This mechanism is consistent with the finding that even single fibres can bulge during fixed-end contraction (Neering et al., 1991).

The stresses in the tissues are defined as the first variation of the strain energy-densities (Equation 2). We need to integrate these stresses in order to obtain the strain-energy potentials from known values of the stress. However, this would only provide a change of the energy; the integral of the stress equals the difference of the strain-energy potentials between two different states. The strain-energy potentials presented in this study are relative to the undeformed state *V*_0_ of the whole muscle.

This paper focusses on the internal energy within the muscle during fixed-end contraction. However, it should be noted that the whole energy balance will also include external work done at the surface of the muscle (Equation 6 in section Appendix I. Details of Model Formulation). For the case of the fixed-end block simulations at the initial muscle length, this external work is zero. However, we had to apply external work to the *x*-faces of the system to lengthen of shorten the muscle blocks for Figure 5B. It should be noted that external work could be done at any point on the muscle surface, for instance transverse compression of the muscle (in the *yz*-plane), and this is the topic of our companion paper. Additionally, kinematic energy is required to accelerate the tissue mass, and should be included to the energy balance (Equation 5 in section Appendix I. Details of Model Formulation) to understand the effect of muscle mass on dynamic contractions of whole muscle: this is the topic of our second companion paper.

### Implications for Muscle Structure and Function

Here we show that considerable strain-energy potential develops in the base material during fixed-end muscle contractions, with this strain-energy potential increasing as muscles become more pennate (Figures 6F, 8D). The base material resists the contractile force in the longitudinal direction, and so the increasing involvement of the base material results in a progressive suppression of the muscle force for more pennate muscle. In this study we have implemented the base material as an isotropic material. However, elements of the base material do have anisotropy that is conferred by their structure such as the network of connected tunnels forming the endomysium that contain a feltwork of collagen fibres (Purslow and Trotter, 1994). Lumped constitutive models, such as used here and by e.g., Blemker et al. (2005) and Rahemi et al. (2015), are unlikely to capture the details of base material anisotropy and the asymmetric response to compression and tension (Böl et al., 2012; Gindre et al., 2013). Anisotropy in the base material properties is most pronounced when the tissue is in tension and may be reasonably disregarded for compressive tests with λ_tot_ < 1 (Böl et al., 2012), as is the case for all the block tests in Figures 4, 5A, 6, 8. Nevertheless, we suggest here that for all the fixed-end tests in this study (regardless of the degree of anisotropy in the base material), the base material would still act to resist muscle deformation and the force in the longitudinal direction; however, as seen by Hodgson et al. (2012), the extent to which the base material interacts with the fibre direction, and thus muscle pennation, depends on the extent of its anisotropy. We additionally show that even though the changes in volumetric strain-energy potential are small, relative to the base material strain-energy potential, the contribution of the volumetric strain-energy potential to the contractile stress and force can be considerable (Figure 5). Thus, even though the contribution of volumetric and base material strain-energy potentials has been largely ignored to date in considerations of whole muscle force and deformation, we suggest that they play an important role in the 3D structure and function of whole-muscle contractions. Subsequently, this finding and study highlight how little we currently know about these processes, and how important it will be to further characterise and implement the base material and volumetric properties of muscle as we continue to learn about 3D function of whole muscle contractions.

The muscle force and stress in the longitudinal direction were reduced at the higher pennation angles (Figures 5A, 8E). This may be partly due to a region of muscle tissue in the middle of the blocks having fibres that did not connect directly to the *x*-faces of the block. These “unsupported” fibres would still develop strain-energy potentials as they deformed their base material during shortening; however, it is possible that lateral transmission of force across the base material was not fully accommodated by the model parameters used, thus these fibres may not have fully contributed to the *F*_*x*_ forces experienced by the *x*-faces of the block. Nonetheless, our results show no evidence that increased pennation angle β_0_ causes an increase in the force in the longitudinal direction of the muscle. Instead, the results from this study support the notion that the functional benefit of pennation in muscle may be to reduce the metabolic cost of contraction (Biewener, 2003), or allowing the fibres to reduce their contractile velocity and thus be better geared for dynamic force production (Azizi et al., 2008), rather than to increase the muscle force for fixed-end contractions *per se* (Alexander, 1983; Lieber and Fridén, 2000; Biewener, 2003).

We have previously shown how intramuscular fat decreases the force and stress that can be produced by contracting muscle (Rahemi et al., 2015), using a similar FEM approach to this current paper. In the fat study (Rahemi et al., 2015) we implemented the intramuscular fat into model simulations in a number of ways and found that all the fatty models generated lower fibre stress and muscle force than their lean counterparts. This effect was due to the higher base material stiffness of the tissue in the fatty models. This fat study highlighted how the material properties of the base material may cause important changes to muscle contractile performance, and this was due to the same mechanisms of energy redistribution as we now describe in this current study. There are a range of muscle conditions and impairments that are associated with increases in fibrotic tissue, changing muscle stiffness, and this energetic framework now provides an approach for us to understand how such conditions lead to loss of muscle function. For instance, altered material properties of muscle tissue post stroke (Lee et al., 2015) and with cerebral palsy (Lieber and Fridén, 2019) have been linked to increases in collagenous connective tissue within the muscle (Lieber and Ward, 2013). Whilst it is possible to measure proxies of muscle tissue stiffness with shear wave ultrasound elastography (Lee et al., 2015), it is difficult to partition these changes between the passive stiffness of the fibres, or the stiffness of the base material. Nonetheless, increased collagen content in the extracellular matrix (that contributes to the base material properties in this study) causes an increase in the passive stiffness of the muscle in mice (Meyer and Lieber, 2011; Wood et al., 2014). As such, we suggest that understanding how altered tissue properties affect the energetic consequences of muscle deformations will allow us to understand muscle impairments in greater detail.

## Conclusions

1. Strain-energy potentials develop within muscle tissue during contraction, even for fixed-end contractions where there is no external work.
2. Strain-energy potentials are distributed across different components within the muscle: the contractile elements as the active- and passive-fibre strain-energy potentials, the cellular and extracellular components as the base material strain-energy potentials and the volumetric component as the volumetric strain-energy potential to enforce the nearly isovolumetric constraints. The balance of this strain-energy distribution may seem counter intuitive, and it depends on the length of the muscle and the orientation of its fibres.
3. The volumetric and base material strain-energy potentials redistribute the energy into all three dimensions and affect the 3D deformations of the muscle. Despite the changes in volumetric strain-energy potential being small, relative to the base material strain-energy potential, the contribution of the volumetric strain-energy potential to the contractile stress and force can be considerable. Even though the contributions of volumetric and base material strain-energy potentials have been largely ignored to date in considerations of whole muscle force and deformation, we suggest that they play an important role in the 3D structure and function of whole-muscle contractions.
4. The muscle volume and girth can change, even for fixed-end contractions, due to the volumetric strain energy potential. This strain energy potential is part of the energy balance, and accounts for the energetic cost of changes in muscle volume.
5. Strain-energy potentials taken up by the volumetric (at shorter muscle lengths) and base material (at short muscle lengths and higher pennation angles) components result in forces that resist the muscle contraction.
6. The active muscle force in the longitudinal direction is thus less than could be predicted from the intrinsic properties of the contractile elements alone. This loss in force gets more pronounced for highly pennate muscle, particularly where β_0_ > 20.
7. There are a range of muscle conditions and impairments that are associated with increases in fibrotic tissue, changing muscle stiffness. The energetic framework that we present in this paper provides an approach for us to understand how changes to the base-material or extracellular properties of a muscle will lead to loss of muscle function.

## Data Availability Statement

The datasets generated for this study are available on request to the corresponding author.

## Ethics Statement

The studies involving human participants were reviewed and approved by University of New South Wales' Human Research Ethics Committee HREC (approval HC17106). The patients/participants provided their written informed consent to participate in this study.

## Author Contributions

JW, SR, DR, SD, and NN contributed to the study design. JW, SR, DR, RK, SD, and NN contributed to the model development. BB conducted the MRI measurements and analysis. SR and BB ran all the simulations for the paper. JW, SR, DR, BB, RK, SD, and NN contributed to the data analysis and manuscript preparation. All authors contributed to the article and approved the submitted version.

## Conflict of Interest

The authors declare that the research was conducted in the absence of any commercial or financial relationships that could be construed as a potential conflict of interest.

## Nomenclature

**Activation** specifically refers to the active *state* of the contractile elements (muscle fibres), and is used to scale the active force that they can develop.

**Muscle contraction** is the *process* of muscle developing forces when its activation level is greater than zero. In muscle physiology, contraction does not necessarily mean shortening because tension can be developed without a change in length.

**Isometric** is a term commonly used in muscle physiology to describe contractions where the length does not change, but this can refer to length at a range of scales, including the sarcomere, muscle belly, muscle-tendon unit or joint angle. We use isometric here to refer to the common physiological measure of maximum isometric force and stress, and also to describe the MRI experiments when the subjects had fixed ankle joint angles.

**Fixed-end** is used to refer to all the muscle block simulations where the blocks had their +*x* and -*x* faces fixed, and the distance between these faces did not change when the activation increased. It is recognised that during these contractions the local fibre stretch varied through the block, and was not always equal to one, and therefore these fixed-end contractions were not isometric at the level of the contractile fibres at each quadrature point.

The **longitudinal direction** is the major *x*-axis of each muscle block. This can be considered the direction that would be between the proximal and distal tendons in a fusiform muscle, and so it is in the commonly phrased ‘line of action’. We do not use ‘line-of-action’ (except when referencing sarcomere properties), because forces and deformations occur in three dimensions in this study and so there is no unique line-of-action.

**Transverse direction** is used to describe directions in the *y-z* plane, and thus is perpendicular to the longitudinal direction of the muscle block.

## Appendices

### Appendix I. Details of Model Formulation

See section 3.1.1. for the general formulation of the finite element model and Table A1 for the definitions and notation of the variables that we used in this section.

**Table A1:** Variables only in appendix.

**Symbol**	**Definition**	**Symbol**	**Definition**
*S*_0_	surface of *V*_0_	${\hat{n}}_{0}$	normal unit vector on *S*
*V*	current configuration	${\hat{a}}_{0}$	normalized fibre orientation in *V*_0_
*S*	surface of *V*	**t**	applied traction
*S*_t_	region of *S* with applied traction	κ_mus_	bulk modulus muscle
*S*_d_	region of *S* with applied displacement	κ_apo_	bulk modulus aponeurosis
*Vol*	current volume	**F**	deformation tensor
*A*	area in *V*	**I**	identity tensor
*t*	time	**B**	left Cauchy tensor
*q*_0_	point in *V*_0_	**B**_**iso**_	isovolumetric left Cauchy tensor
*q*	point in *V*	**σ**	Cauchy stress tensor
*U*_vol_	volumetric strain-energy potential	**τ**	Kirchhoff tensor
*U*_iso_	isovolumetric strain-energy potential	*I*_1_	first invariant
*U*_base_	base material strain-energy potential	*I*_3_	third invariant
*U*_fibre_	fibre strain-energy potential	*I*_4_	fourth invariant
*U*_apo,base_	aponeurosis base material strain-energy potential	â	activation level in muscle fibres
*U*_apo,fibre_	aponeurosis fibre strain-energy potential	σ_0_	maximum isometric stress of contractile elements
*U*_mus,base_	muscle base material strain-energy potential	σ_base_	base material stress
*U*_mus,fibre_	muscle fibre strain-energy potential	σ_mus,base_	muscle base material stress
*U*_act_	active muscle fibre strain-energy potential	σ_apo,base_	aponeurosis base material stress
*U*_pas_	passive muscle fibre strain-energy potential	σ_fibre_	fibre stress
ψ_vol_	volumetric strain energy-density	σ_mus,fibre_	muscle fibre stress
ψ_iso_	isovolumetric energy-density	σ_apo,fibre_	aponeurosis fibre stress
ψ_base_	base energy-density	${\hat{\sigma }}_{\text{act}}$	active muscle fibre stress
ψ_fibre_	fibre energy-density	${\hat{\sigma }}_{\text{pas}}$	passive muscle fibre stress
ψ_apo,base_	aponeurosis base material energy-density	∇_0_	gradient with respect to *V*_0_
ψ_apo,fibre_	aponeurosis fibre energy-density	∇	gradient with respect to *V*
ψ_mus,base_	muscle base material energy-density	**div**	tensorial divergence with respect to *V*
ψ_mus,fibre_	muscle fibre energy-density	**0**	zero vector

The Cauchy stress in the tissues σ is defined as the first variation of the internal strain energy-density ψ_int_ (see Equation 3). To obtain the strain-energy potentials, we integrate the Cauchy stress. However, this provides a change of the energy; the integral of the stress equals the difference of the strain-energy potential between two different states (see Equation 7). The strain-energy potentials reported in this study are relative to the undeformed state *V*_0_ of the whole muscle. In our formulation, the undeformed configuration has a zero strain-energy potential as all internal stresses and external forces in the system are zero.

This paper focuses on the change of the internal strain-energy potential *U*_int_ within the muscle during fixed-end contraction. However, it should be noted that the whole energy balance will also include external work done at the surface of the muscle (Equation 6). For the case of the fixed-end block simulations at the initial muscle length, this external work is zero. However, when the muscle block was initially stretched or shortened to a new passive length, with traction being applied to the positive *x*-face, external work is added to the system.

We assume that a muscle occupies an initial configuration *V*_0_ and its surface is *S*_0_. Using the principles of continuum mechanics, a point *q*_0_ in *V*_0_ can be tracked over time after the muscle in *V*_0_ has seen some deformation. We consider *V* as the current configuration, where the muscle in *V*_0_ has been deformed at time *t*. We assume that there exists a unique point *q* in *V* such that *q* = *q*(*q*_0_*,t*). This means that the point *q* is the representation of the point *q*_0_ in the current configuration of the muscle. The displacement vector at the point *q*_0_ in *V*_0_ is defined as the vector formed by the points *q* and *q*_0_, that is

$$\text{u}\left(q_{0},t\right)\;:=q\left(q_{0},t\right)-q_{0}.\;$$

The deformation of the tissues is defined as the gradient of this displacement. This deformation is quantified as a tensor of infinitesimal changes of points *q*_0_ in *V*_0_ at time *t*. Mathematically, this is defined as:

$$\text{F }:=\text{I}+\nabla_{0}\text{u},\;$$

where **I** is the 3-by-3 identity matrix and ∇_0_ is the gradient of a vector with respect to *V*_0_. We denote the dilation of the tissues by *J*, and the internal pressure in the tissues by *p*.

The deformation tensor **F** provides a change of variables for integrals over *V*_0_ and *V* as well as over the surfaces *S*_0_ and *S*. The following change of variables hold:

$$dV=I_{3}\left(\text{F}\right)dV_{0},\;\hat{\text{n}}dS=I_{3}\left(\text{F}\right){\text{F}}^{-t}{\hat{\text{n}}}_{0}dS_{0},\;$$

where $\hat{\text{n}}$ is the unit normal vector on *S* and $\hat{\text{n}}$_0_ is the unit normal vector on *S*_0_, and **F**^−*t*^ is the transpose tensor of the inverse tensor of the deformation tensor, **F**^−1^. These changes of variables are used to compute volume and area of the deformed configurations of the muscle. We compute these as follows:

$$\text{Vol }:=\int_{V_{0}}I_{3}\left(\text{F}\right)dV_{0},\;A\;:=\int_{S_{0}}I_{3}\left(\text{F}\right)\sqrt{{\hat{\text{n}}}_{0}^{t}{\text{F}}^{-1}{\text{F}}^{-t}{\hat{\text{n}}}_{0}}dS_{0}.$$

The fourth invariant *I*_4_ of **B**_iso_:=*I*_3_(**B**)^−1/3^ **B**, where **B** is the left Cauchy tensor defined as **B** := **F**^*t*^**F**, is used in our model to represent the isovolumetric stretch of the fibres at the point *q*_0_ in *V*_0_, denoted by λ_iso_. Following the ideas in Simo et al. (1985), we consider normalized vectors **â**_0_ = **â**_0_(*q*_0_) that are tangential to the fibres at the point *q*_0_ in *V*_0_. We refer to **â**_0_ as the initial orientation of the fibre at the point *q*_0_ in *V*_0_. The total stretch of the fibres at *q*_0_ in *V*_0_ is denoted by λ_tot_ and is defined as:

$$\lambda_{\text{tot}}\;:=I_{3}{\left(\text{F}\right)}^{1/3}\lambda_{\text{iso}},\;\lambda_{\text{iso}}=I_{4}\left({\hat{\text{a}}}_{0},{\text{B}}_{\text{iso}}\right).\;$$

Important elasticity tensors can be obtained from the internal strain energy-density ψ_int_. The Kirchhoff tensor **τ** and the Cauchy tensor **σ** are defined as:

(3) $$\begin{array}{l} \tau \;:=2\text{B}\frac{\partial \psi_{\text{int}}}{\partial \text{B}},\;\sigma \;:=I_{3}{\left(\text{F}\right)}^{-1}\tau . \end{array}$$

The strain energy-density ψ_int_ is split into volumetric and isovolumetric strain energy-densities ψ_vol_ and ψ_iso_. Following (Pelteret and McBride, 2012), the volumetric strain energy-density ψ_vol_ is defined as:

$$\psi_{\text{vol}}\left(\text{u},\;p,J\right)\;:=\frac{\kappa }{4}\left(J^{2}-2\log\left(J\right)-1\right)+p\left(J-I_{3}\left(\text{F}\right)\right),$$

where κ > 0 is the bulk modulus of the tissue. The value of the bulk modulus is different for muscle and aponeurosis, κ_mus_ = 10^6^ and κ_apo_ = 10^8^ (see Rahemi et al., 2014). We add the pressure *p* as a Lagrange multiplier in our system to ensure an accurate computation of the dilation *J*.

The isovolumetric strain energy-density is the sum of the base material and fibre strain energy-densities:

$${\text{Ψ}}_{\text{iso}}\left(\text{u}\right)\;:={\text{Ψ}}_{\text{base}}\left(I_{1}\left({\text{B}}_{\text{iso}}\right)\right)+{\text{Ψ}}_{\text{fibre}}\left(\lambda_{\text{iso}}\right).\;$$

We fit parameters from the models proposed by Yeoh (1993) to data reported in Mohammadkhah et al. (2016) and Azizi et al. (2009) to obtain the mechanical properties for the base material in both muscle and aponeurosis, respectively (see section 3.1.2). The strain energy-density ψ_base_ and the scalar stress σ_base_ satisfy the following:

$$\frac{\partial {\text{Ψ}}_{\text{base}}}{\partial I_{1}}=\sigma_{\text{base}}\left(I_{1}\left({\text{B}}_{\text{iso}}\right)\right).\;$$

The muscle base material properties differ widely from those of the aponeurosis. We use ψ_mus,base_ and σ_mus,base_ for muscle tissues, and use ψ_apo,base_ and σ_apo,base_ for aponeurosis tissues.

Following Blemker et al. (2005), the scalar stress in the fibres σ_fibre_ is related to the strain energy-density ψ_fibre_ as follows:

(4) $$\begin{array}{l} \lambda_{\text{iso}}\frac{\partial \psi_{\text{fibre}}}{\partial \lambda_{\text{iso}}}=\sigma_{\text{fibre}}\left(\lambda_{\text{iso}}\right) \end{array}$$

For muscle tissues, passive and active properties are part of the normalized fibre stress $\hat{\sigma }$. We also consider the activation of the tissues, denoted by â = â(*q*_0_, *t*), and assume it only affects the active stresses. The stress due to fibre stretch in muscle fibres is defined as

$${\hat{\sigma }}_{\text{mus},\text{fibre}}\left(\lambda_{\text{iso}}\right):=â\left(q_{0},t\right){\hat{\sigma }}_{\text{act}}\left(\lambda_{\text{iso}}\right)+{\hat{\sigma }}_{\text{pas}}\left(\lambda_{\text{iso}}\right),\;$$

where $\hat{\sigma }$_act_ represents the stress in the tissues due to active stretch of the muscle fibres and $\hat{\sigma }$_pas_ is the stress in the tissues due to passive stretch of the muscle fibres. Altogether, we can relate the stresses in the muscle fibres to the strain energy-density ψ_mus,fibre_ by using Equation (4):

$$\lambda_{\text{iso}}\frac{\partial \psi_{\text{mus},\text{fibre}}}{\partial \lambda_{\text{iso}}}=\;\sigma_{0}{\hat{\sigma }}_{\text{mus},\text{fibre}}\left(\lambda_{\text{iso}}\right),$$

where σ_0_ is the maximum isometric stress of the contractile elements.

For aponeuroses, the stresses in the fibres are only due to passive stretch of the fibres. The strain energy-density ψ_apo,fibre_ and the scalar stress in the aponeurosis fibres σ_apo,fibre_ are defined exactly as in Equation (4):

$$\lambda_{\text{iso}}\frac{\partial \psi_{\text{apo},\text{fibre}}}{\partial \lambda_{\text{iso}}}=\;\sigma_{\text{apo},\text{fibre}}\left(\lambda_{\text{iso}}\right).$$

Our approach seeks a displacement **u**, an internal pressure *p* and a dilation *J* which minimize the total energy of the system. Let *E*_tot_ be the total strain energy in the muscle. Under the assumption of a quasi-static regime, the total energy can be considered as the sum of the internal strain-energy potential and the work done on the system by external forces. Given an applied traction **t** on a part of the surface *S* of *V*, denoted by *S*_t_, the total strain energy of a deformed muscle in *V* can be defined as:

(5) $$\begin{array}{l} E_{\text{tot}}\left(\text{u},p,J\right)=U_{\text{int}}\left(\text{u},p,J\right)-W_{\text{ext}}\left(\text{u}\right), \end{array}$$

where

(6) $$\begin{array}{l} U_{\text{int}}\left(\text{u},\;p,J\right)\;:=\int_{V}{\text{Ψ}}_{\text{int}}\left(\text{u},p,J\right)dV. \end{array}$$

We utilize the finite element method to approximate states (**u***, p, J*) at which the first variation *DE*_tot_ of the total strain energy is zero. That is, we numerically solve the following equation for the unknowns (**u***, p, J*):

$$DE_{\text{tot}}\left(\text{u},p,J\right)=0.\;$$

The first variation of the total strain energy gives the following set of equations:

$$\begin{array}{l} \text{div}\sigma \left(\text{u}\right)=0,J-I_{3}\left(\text{u}\right)=0,p-\frac{\kappa }{2}\left(J-\frac{1}{J}\right)=0\text{ in }V,\\ \sigma \left(\text{u}\right)\hat{n}=t\text{ on }S_{\text{t}},\text{u}=0\text{ on }S_{\text{d}}, \end{array}$$

where the vector $\hat{\text{n}}$ denotes the unit normal vector on *S*, and *S*_d_ stands for the part of *S* on which the displacement **u** is prescribed. Note that in our model we clamp the displacement **u** on *S*_d_.

The change in the internal strain-energy potential reported in this paper is computed using the following definition:

(7) $$\begin{array}{l} U_{\text{int}}\left(\text{u},\;p,J\right)\;:=\int_{V_{0}}\tau :\nabla \text{u}\;dV_{0}+U_{\text{int}}\left({\text{u}}_{0},p_{0},J_{0}\right), \end{array}$$

where (**u**_0_*,p*_0_*,J*_0_) is a known state of the displacement, pressure and dilation. We compute the volumetric, isovolumetric, muscle base material, aponeurosis base material, muscle active-fibre, muscle passive-fibre, and aponeurosis fibre strain-energy potentials *U*_vol_, *U*_iso_, *U*_mus,base_, *U*_apo,base_, *U*_act_, *U*_pas_, and *U*_apo,fibre_ respectively by using the formula given in Equation (7):

$$U_{\text{vol}}\left(\text{u},\;p,J\right)\;:=\int_{V_{0}}pJ\text{I}:\nabla \text{u}\;dV_{0}+U_{\text{vol}}\left({\text{u}}_{0},\;p_{0},J_{0}\right),\;$$

$$U_{\text{iso}}\left(\text{u}\right)\;:=2\int_{V_{0}}\text{B}\frac{\partial {\text{Ψ}}_{\text{iso}}}{\partial \text{B}}:\nabla \text{u}\;dV_{0}+U_{\text{iso}}\left({\text{u}}_{0},\;p_{0},J_{0}\right),\;$$

$$\begin{array}{l} U_{\text{mus},\text{base}}\left(\text{u}\right)\;:=2\int_{V_{0}}\sigma_{\text{mus},\text{base}}\left(I_{1}\right)\text{B}\frac{\partial I_{1}}{\partial \text{B}}:\nabla \text{u}\;dV_{0}+\\ \;U_{\text{mus},\text{base}}\left({\text{u}}_{0},\;p_{0},J_{0}\right), \end{array}$$

$$\begin{array}{l} U_{\text{apo},\text{base}}\left(\text{u}\right)\;:=2\int_{V_{0}}\sigma_{\text{apo},\text{base}}\left(I_{1}\right)\text{B}\frac{\partial I_{1}}{\partial \text{B}}:\nabla \text{u}\;dV_{0}+\\ \;U_{\text{apo},\text{base}}\left({\text{u}}_{0},\;p_{0},J_{0}\right), \end{array}$$

$$U_{\text{act}}\left(\text{u}\right):=\sigma_{0}\int_{V_{0}}\frac{{\hat{\sigma }}_{\text{act}}\left(\lambda_{\text{iso}}\right)}{\lambda_{\text{iso}}}\text{B}\frac{\partial I_{1}}{\partial \text{B}}:\nabla \text{u}\;dV_{0}+U_{\text{act}}\left({\text{u}}_{0},\;p_{0},J_{0}\right),\;$$

$$U_{\text{pas}}\left(\text{u}\right):=\sigma_{0}\int_{V_{0}}\frac{{\hat{\sigma }}_{\text{pas}}\left(\lambda_{\text{iso}}\right)}{\lambda_{\text{iso}}}\text{B}\frac{\partial I_{1}}{\partial \text{B}}:\nabla \text{u}\;dV_{0}+U_{\text{pas}}\left({\text{u}}_{0},\;p_{0},J_{0}\right),\;$$

$$\begin{array}{l} U_{\text{apo},\text{fibre}}\left(\text{u}\right)\;:=\sigma_{0}\int_{V_{0}}\frac{\sigma_{\text{apo},\text{fibre}}\left(\lambda_{\text{iso}}\right)}{\lambda_{\text{iso}}}\text{B}\frac{\partial I_{1}}{\partial \text{B}}:\nabla \text{u}\;dV_{0}\\ \;+U_{\text{apo},\text{fibre}}\left({\text{u}}_{0},\;p_{0},J_{0}\right). \end{array}$$

We assume that **u**_0_, *p*_0_, and *J*_0_ are the displacement, pressure, and dilation in *V*_0_. In our simulations we set the displacement **u**_0_ to be the zero vector **0**, the pressure *p*_0_ to be zero, and the dilation *J*_0_ to be 1. The definition of the intrinsic properties of the tissues implies that all the strain-energy potentials in the equations above at the state (**u**_0_*, p*_0_*, J*_0_) vanish.

### Appendix II. Experimental Measurements From MRI and DTI

This appendix describes experimental data collection and analysis for measurement of the changes in three-dimensional whole-muscle shape and muscle fibre orientation of the human medial gastrocnemius during fixed-end (constant ankle angle) plantarflexion contractions.

#### Data Acquisition

We obtained mDixon magnetic resonance imaging (MRI) and diffusion tensor imaging (DTI) scans of the right lower legs of four female participants (age 29 ± 4 years mean ± S.D.). All procedures conformed to the Declaration of Helsinki (2008) and were approved by University of New South Wales' Human Research Ethics Committee HREC (approval HC17106). Written informed consent was obtained from all participants.

Participants lied supine with the knee slightly flexed in a 3T MRI scanner (Philips Achieva TX, Best, the Netherlands). We used a cardiac coil with 32 elements for receive. The anterior part of the coil was placed on the tibia and the posterior part of the coil was placed on the MRI table. The knee was supported by a foam wedge to maintain a small gap between the coil and the posterior calf. The right foot was strapped tightly into a footplate, which was connected to a custom-built MRI-compatible force transducer. The ankle was held in 5° plantarflexion relative to the neutral position with the foot perpendicular to the tibia. MRI and DTI scans were obtained while the participant's muscles were relaxed (rest) and during plantarflexion contractions at 10% (twice) and 20% (once) of their maximum voluntary isometric plantarflexion torque, which was previously determined with a dynamometer. We chose scan sequences and fields of view to have a maximal scan duration of 2.5 min, because pilot testing showed that all participants could maintain 20% plantarflexion torque for up to 2.5 min while staying sufficiently still to obtain high quality MRI scans. mDixon and DTI scans were obtained during separate contractions with participants given at least two minutes rest in between scans. The scans did not cover the most proximal part (~5 cm) of the medial gastrocnemius. Visual feedback was provided on a monitor next to the MRI bed to help participants maintain the target force during the scans.

The settings of the mDixon scan were: 2-point 3D mDixon FFE, TR/TE1/TE2 6/3.5/4.6 ms, field of view 180 × 180 mm, 250 slices, slice thickness 1 mm, acquisition matrix 180 × 180 (reconstructed to 192 × 192), reconstructed voxel size 0.94 × 0.94 × 1 mm, number of signal averages 1 and scan time 138 s. The settings of the DTI scan were: DT-EPI with spectral pre-saturation with inversion recovery (SPIR) fat suppression, TR/TE 3836/63 ms, field of view 180 × 180 mm, 40 slices, slice thickness 5 mm, 16 gradient directions on a hemisphere, number of signal averages (NSA) = 1 for diffusion-weighted images and NSA = 2 for the b0 image, *b* = 500 s/mm^2^ (b0 image with *b* = 0 s/mm^2^), diffusion gradient time Δ/δ = 30.4/8.2 msec and scan time 134 s.

#### Medial Gastrocnemius 3D Surface Models

We created 3D surface models of the medial gastrocnemius muscle at rest by manually outlining the boundary of the medial gastrocnemius on the water image of the mDixon scan (Bolsterlee et al., 2017; Figure 2). We used non-rigid registration algorithms (Elastix 4.7; Klein et al., 2010) to create surface models of the deformed muscles during contractions and visually inspected the results to determine that the deformed outlines followed closely the boundaries of the muscle on the scans obtained during contractions. The surface models were rotated to a local coordinate system using principal component analysis on the vertices (nodes) of the surface model so that the *x*-axis aligns with the long axis and the *y*- and *z*-axis with the width and depth axes, respectively.

#### Fibre Orientation at Rest and During Contractions

We determined muscle fibre orientations from the DTI scans (Bolsterlee et al., 2019), assuming that the primary eigenvector of the diffusion tensor aligned with muscle fibre orientations (Damon et al., 2002). First, DTI scans were filtered using a local principal component analysis filter (Manjon et al., 2013), after which the diffusion tensor was reconstructed using DSI Studio (Yeh et al., 2013). The fibre orientation of a voxel was defined as the angle in 3D between the primary eigenvector of the diffusion tensor and the *x*-axis (longitudinal axis) of the muscle. We calculated the fibre orientation of the muscle at rest and during contractions as the average fibre orientation of voxels inside a 30 × 10 × 10 mm region sampled in the middle of the muscle (Figure 2).

## References

[B1] Aidley D. J. (1998). The Physiology of Excitable Cells, 4th Edn Cambridge: Cambridge University Press.

[B2] Alexander R. M. (1983). Animal Mechanics, 2nd Edn Oxford: Blackwell Scientific Publications Ltd.

[B3] Alipour M. Mithraratne K. Fernandez J. (2017). A diffusion-weighted imaging informed continuum model of the rabbit triceps surae complex. Biomech. Model. Mechanobiol. 16, 1729–1741. 10.1007/s10237-017-0916-4 28516387

[B4] Arndt D. Bangerth W. Davydov D. Heister T. Heltai L. Kronbichler M. (2017). The deal.II library, version 8.5. J. Numer. Math. 25, 137–145. 10.1515/jnma-2017-0058

[B5] Azizi E. Brainerd E. Roberts T. (2008). Variable gearing in pennate muscles. Proc. Natl. Acad. Sci. U.S.A. 105, 1745–1750. 10.1073/pnas.0709212105 18230734 PMC2234215

[B6] Azizi E. Deslauriers A. R. Holt N. C. Eaton C. E. (2017). Resistance to radial expansion limits muscle strain and work. Biomech. Model. Mechanobiol. 16, 1633–1643. 10.1007/s10237-017-0909-3 28432448 PMC5889119

[B7] Azizi E. Halenda G. M. Roberts T. J. (2009). Mechanical properties of the gastrocnemius aponeurosis in wild turkeys. Integr. Comp. Biol. 49, 51–58. 10.1093/icb/icp006 21120110 PMC2994030

[B8] Barnes W. S. (1986). The relationship between maximum isometric strength and intramuscular circulatory occlusion. Ergonomics 23, 351–357. 10.1080/00140138008924748 7202390

[B9] Biewener A. A. (2003). Animal Locomotion. Oxford: Oxford University Press.

[B10] Blemker S. S. Pinsky P. M. Delp S. L. (2005). A 3D model of muscle reveals the causes of nonuniform strains in the biceps brachii. J. Biomech. 38, 657–665. 10.1016/j.jbiomech.2004.04.009 15713285

[B11] Böl M. Kruse R. Ehret A. E. Leichsenring K. Siebert T. (2012). Compressive properties of passive skeletal muscle—the impact of precise sample geometry on parameter identification in inverse finite element analysis. J. Biomech. 45, 2673–2679. 10.1016/j.jbiomech.2012.08.023 22954714

[B12] Böl M. Leichsenring K. Weichert C. Sturmat M. Schenk P. Blickhan R.. (2013). Three-dimensional surface geometries of the rabbit soleus muscle during contraction: input for biomechanical modelling and its validation. Biomech. Model. Mechanobiol. 12, 1205–1220. 10.1007/s10237-013-0476-1 23417261

[B13] Böl M. Reese S. (2008). Micromechanical modelling of skeletal muscles based on the finite element method. Comp. Meth. Biomech. Biomed. Eng. 11, 489–504. 10.1080/10255840701771750 19230146

[B14] Böl M. Sturmat M. Weichert C. Kober C. (2011). A new approach for the validation of skeletal muscle modelling using MRI data. Comput. Mech. 47, 591–601. 10.1007/s00466-010-0567-0

[B15] Bolsterlee B. D'Souza A. Gandevia S. C. Herbert R. D. (2017). How does passive lengthening change the architecture of the human medial gastrocnemius muscle? J. Appl. Physiol. 122, 727–738. 10.1152/japplphysiol.00976.2016 28104754

[B16] Bolsterlee B. D'Souza A. Herbert R. D. (2019). Reliability and robustness of muscle architecture measurements obtained using diffusion tensor imaging with anatomically constrained tractography. J. Biomech. 86, 71–78. 10.1016/j.jbiomech.2019.01.043 30739766

[B17] Brainerd E. L. Azizi E. (2005). Muscle fiber angle, segment bulging and architectural gear ratio in segmented musculature. J. Exp. Biol. 208, 3249–3261. 10.1242/jeb.01770 16109887

[B18] Chen J. S. Basava R. R. Zhang Y. Csapo R. Malis V. Sinha U.. (2016). Pixel-based meshfree modelling of skeletal muscles. Comput. Methods Biomech. Biomed. Eng. Imaging Vis. 4, 73–85. 10.1080/21681163.2015.1049712 28748126 PMC5523135

[B19] Chi S. W. Hodgson J. Chen J. S. Reggie Edgerton V. Shin D. D. Roiz R. A.. (2010). Finite element modeling reveals complex strain mechanics in the aponeuroses of contracting skeletal muscle. J. Biomech. 43, 1243–1250. 10.1016/j.jbiomech.2010.01.005 20189180 PMC2857665

[B20] Cobb M. (2002). Exorcizing the animal spirits: Jan Swammerdam on nerve function. Nat. Rev. Neurosci. 3, 395–400. 10.1038/nrn806 11988778

[B21] Damon B. M. Ding Z. H. Anderson A. W. Freyer A. S. Gore J. C. (2002). Validation of diffusion tensor MRI-based muscle fiber tracking. Magn. Reson. Med. 48, 97–104. 10.1002/mrm.10198 12111936

[B22] de Brito Fontana H. Han S. W. Sawatsky A. Herzog W. (2018). The mechanics of agonistic muscles. J. Biomech. 79, 15–20. 10.1016/j.jbiomech.2018.07.007 30195849

[B23] Dick T. J. M. Arnold A. S. Wakeling J. M. (2016). Quantifying Achilles tendon force *in vivo* from ultrasound images. J. Biomech. 49, 3200–3207. 10.1016/j.jbiomech.2016.07.036 27544621 PMC5074891

[B24] Dick T. J. M. Wakeling J. M. (2017). Shifting gears: dynamic muscle shape changes and force-velocity behavior in the medial gastrocnemius. J. Appl. Physiol. 123, 1433–1442. 10.1152/japplphysiol.01050.2016 28860176 PMC5814684

[B25] Gans C. Bock W. (1965). The functional significance of muscle architecture – a theoretical analysis. Adv. Anat. Embryol. Cell Biol. 38, 115–142. 5319094

[B26] Gardiner J. C. Weiss J. A. (2000). Simple shear testing of parallel-fibered planar soft tissues. J. Biomech. Eng. 123, 170–175. 10.1115/1.1351891 11340878

[B27] Gindre J. Takaza M. Moerman K. M. Simms C. K. (2013). A structural model of passive skeletal muscle shows two reinforcement processes in resisting deformation. J. Mech. Behav. Biomed. Mater. 22, 84–94. 10.1016/j.jmbbm.2013.02.007 23587721

[B28] Gregorio C. C. Granzier H. Sorimachi H. Labeit S. (1999). Muscle assembly: a titanic achievement? Curr. Opin. Cell Biol. 11, 18–25. 10.1016/S0955-0674(99)80003-9 10047523

[B29] Günther M. Röhrle O. Haeufle D. F. B. Schmitt S. (2012). Spreading out muscle mass within a Hill type model: a computer simulation study. Comput. Math. Methods Med. 2012:848630. 10.1155/2012/848630 23227110 PMC3512296

[B30] Héroux M. E. Stubbs P. W. Herbert R. D. (2016). Behavior of human gastrocnemius muscle fascicles during ramped submaximal isometric contractions. Physiol. Rep. 4:e12947. 10.14814/phy2.12947 27604399 PMC5027354

[B31] Higham T. Biewener A. Wakeling J. M. (2008). Functional diversification within and between muscle synergists during locomotion. Biol. Lett. 4, 41–44. 10.1098/rsbl.2007.0472 17986428 PMC2412925

[B32] Hill A. V. (1938). The heat of shortening and the dynamic constants of muscle. Proc. R. Soc. B 126, 136–195. 10.1098/rspb.1938.005016709736

[B33] Hodgson J. A. Chi S. W. Yang J. P. Chen J. S. Edgerton V. R. Sinha S. (2012). Finite element modeling of passive material influence on the deformation and force output of skeletal muscle. J. Mech. Behav. Biomed. Mater. 9, 163–183. 10.1016/j.jmbbm.2012.01.010 22498294 PMC4075494

[B34] Hodson-Tole E. F. Wakeling J. M. Dick T. J. M. (2016). Passive muscle-tendon unit gearing is joint dependant in human medial gastrocnemius. Front. Physiol. 7:95. 10.3389/fphys.2016.00095 27014093 PMC4791406

[B35] Johansson T. Meier P. Blickhan R. (2000). A finite element model for the mechanical analysis of skeletal muscles. J. Theor. Biol. 206, 131–149. 10.1006/jtbi.2000.2109 10968943

[B36] Karakuzu A. Pamuk U. Ozturk C. Acar B. Yucesoy C. A. (2017). Magnetic resonance and diffusion tensor imaging analyses indicate heterogeneous strains along human medial gastrocnemius fascicles caused by submaximal plantar-flexion activity. J Biomech. 57:6978. 10.1016/j.jbiomech.2017.03.028 28433388

[B37] Kawakami Y. Ichinose Y. Fukunaga T. (1998). Architectural and functional features of human triceps surae muscles during contraction. J. Appl. Physiol. 85, 398–404 10.1152/jappl.1998.85.2.398 9688711

[B38] Klein S. Staring M. Murphy K. Viergever M. A. Pluim J. P. (2010). elastix: a toolbox for intensity-based medical image registration. IEEE Trans. Med. Imaging 29, 196–205. 10.1109/TMI.2009.2035616 19923044

[B39] Lee S. S. M. Spear S. Rymer W. Z. (2015). Quantifying changes in material properties of stroke-impaired muscle. Clin. Biomech. 30, 269–275. 10.1016/j.clinbiomech.2015.01.004 25638688 PMC7057856

[B40] Lieber R. L. Fridén J. (2000). Functional and clinical significance of skeletal muscle architecture. Muscle Nerve 23, 1647–1666. 10.1002/1097-4598(200011)23:11<1647::aid-mus1>3.0.co;2-m 11054744

[B41] Lieber R. L. Fridén J. (2019). Muscle contracture and passive mechanics in cerebral palsy. J. Appl. Physiol. 126, 1492–1501. 10.1152/japplphysiol.00278.2018 30571285 PMC6589815

[B42] Lieber R. L. Ward S. R. (2013). Cellular mechanisms of tissue fibrosis. 4. Structural and functional consequences of skeletal muscle fibrosis. Am. J. Physiol. 305, C241–C252. 10.1152/ajpcell.00173.2013 23761627 PMC3742845

[B43] Liu G. R. Quek S. S. (2014). The Finite Element Method, 2nd Edn Oxford: Butterworth-Heinemann.

[B44] Maganaris C. Baltzopoulos V. Sargeant A. (1998). *In vivo* measurements of the triceps surae complex architecture in man: implications for muscle function. J. Physiol. 512, 603–614. 10.1111/j.1469-7793.1998.603be.x 9763648 PMC2231202

[B45] Manjon J. V. Coupe P. Concha L. Buades A. Collins D. L. Robles M. (2013). Diffusion weighted image denoising using overcomplete local PCA. PlOS ONE 8:e73021. 10.1371/journal.pone.0073021 24019889 PMC3760829

[B46] MATLAB (2018). version 9.4.0 (R2018a). Natick, MA: The MathWorks Inc.

[B47] Meier P. Blickhan R. (2000). FEM-simulation of skeletal muscle: the influence of inertia during activation and deactivation, in Skeletal Muscle Mechanics: From Mechanisms to Function, ed Herzog W. (Chichester: John Wiley and Sons Ltd), 209–222.

[B48] Meyer G. A. Lieber R. L. (2011). Elucidation of extracellular matrix mechanics from muscle fibers and fiber bundles. J. Biomech. 44, 771–773. 10.1016/j.jbiomech.2010.10.044 21092966 PMC3042517

[B49] Moerman K. M. (2018). GIBBON: The Geometry and Image-Based Bioengineering add-On. J. Open Source Softw. 3:506 10.21105/joss.00506

[B50] Mohammadkhah M. Murphy P. Simms C. K. (2016). The *in vitro* passive elastic response of chicken pectoralis muscle to applied tensile and compressive deformation. J. Mech. Behav. Biomed. Mater. 62, 468–480. 10.1016/j.jmbbm.2016.05.021 27281164

[B51] Neering I. R. Quesenberry L. A. Morris V. A. Taylor S. R. (1991). Nonuniform volume changes during muscle contraction. Biophys. J. 59, 926–933. 10.1016/S0006-3495(91)82306-2 2065192 PMC1281259

[B52] Oomens C. W. Maenhout M. vanOijen C. H. Drost M. R. Baaijens F. P. (2003). Finite element modelling of contracting skeletal muscle. Philos. Trans. R. Soc. B 358, 1453–1460. 10.1098/rstb.2003.1345 14561336 PMC1693246

[B53] Pamuk U. Karakuzu A. Ozturk C. Acar B. Yucesoy C. A. (2016). Combined magnetic resonance and diffusion tensor imaging analyses provide a powerful tool for *in vivo* assessment of deformation along human muscle fibers. J. Mech. Behav. Biomed. Mater. 63, 207–219. 10.1016/j.jmbbm.2016.06.031 27429070

[B54] Pappas G. P. Asakawa D. S. Delp S. L. Zajac F. E. Drace J. E. (2002). Nonuniform shortening in the biceps brachii during elbow flexion. J. Appl. Physiol. 92, 2381–2389. 10.1152/japplphysiol.00843.2001 12015351

[B55] Pelteret J. P. McBride A. (2012). The Deal.II Tutorial Step-44: Three-field Formulation for Non-linear Solid Mechanics. 10.5281/zenodo.439772

[B56] Purslow P. Trotter J. A. (1994). The morphology and mechanical properties of endomysium in series fibred muscles: variations with muscle length. J. Musc. Res. Cell. M. 15, 299–308. 10.1007/BF00123482 7929795

[B57] Rahemi H. Nigam N. Wakeling J. M. (2014). Regionalizing muscle activity causes changes to the magnitude and direction of the force from whole muscles – a modeling study. Front. Physiol. 5:298. 10.3389/fphys.2014.00298 25232341 PMC4152886

[B58] Rahemi H. Nigam N. Wakeling J. M. (2015). The effect of intramuscular fat on skeletal muscle mechanics: implications for the elderly and obese. J. R. Soc. Interface 12:20150364. 10.1098/rsif.2015.0365 26156300 PMC4535407

[B59] Raiteri B. J. Cresswell A. G. Lichtwark G. A. (2016). Three-dimensional geometrical changes of the human tibialis anterior muscle and its central aponeurosis measured with three-dimensional ultrasound during isometric contractions. PeerJ. 4:e2260. 10.7717/peerj.2260 27547566 PMC4974924

[B60] Rana M. Hamarneh G. Wakeling J. M. (2013). 3D fascicle orientations in triceps surae. J. Appl. Physiol. 115, 116–125. 10.1152/japplphysiol.01090.2012 23640593

[B61] Randhawa A. Jackman M. E. Wakeling J. M. (2013). Muscle gearing during isotonic and isokinetic movements in the ankle plantarflexors. Eur. J. Appl. Physiol. 113, 437–447. 10.1007/s00421-012-2448-z 22777499

[B62] Randhawa A. Wakeling J. M. (2015). Multidimensional models for predicting muscle structure and fascicle pennation. J. Theor. Biol. 382, 57–63. 10.1016/j.jtbi.2015.06.001 26073723

[B63] Randhawa A. Wakeling J. M. (2018). Transverse anisotropy in the deformation of the muscle during dynamic contractions. J. Exp. Biol. 221:jeb175794. 10.1242/jeb.175794 29844202

[B64] Roberts T. J. Eng C. M. Sleboda D. A. Holt N. C. Brainerd E. L. Stover K. K.. (2019). The multi-scale, three-dimensional nature of skeletal muscle contraction. Physiology 34, 402–408. 10.1152/physiol.00023.2019 31577172 PMC7002870

[B65] Röhrle O. Pullan A. J. (2007). Three-dimensional finite element modelling of muscle forces during mastication. J. Biomech. 40, 3363–3372. 10.1016/j.jbiomech.2007.05.011 17602693

[B66] Ross S. A. Nigam N. Wakeling J. M. (2018a). A modelling approach for exploring muscle dynamics during cyclic contractions. PLoS Comp. Biol. 14:e1006123. 10.1371/journal.pcbi.1006123 29659583 PMC5919698

[B67] Ross S. A. Ryan D. S. Dominguez S. Nigam N. Wakeling J. M. (2018b). Size, history-dependent, activation and three-dimensional effects on the work and power produced during cyclic muscle contractions. Integr. Comp. Biol. 58, 232–250. 10.1093/icb/icy021 29726964 PMC6104705

[B68] Ross S. A. Wakeling J. M. (2016). Muscle shortening velocity depends on tissue inertia and level of activation during submaximal contractions. Biol. Lett. 12:20151041. 10.1098/rsbl.2015.1041 27354711 PMC4938035

[B69] Ryan D. S. Stutzig N. Siebert T. Wakeling J. M. (2019). Passive and dynamic muscle architecture during transverse loading for gastrocnemius medialis in man. J. Biomech. 86, 160–166. 10.1016/j.jbiomech.2019.01.054 30792071

[B70] Siebert T. Günther M. Blickhan R. (2012). A 3D-geometric model for the deformation of a transversally loaded muscle. J. Theor. Biol. 298, 116–121. 10.1016/j.jtbi.2012.01.009 22251888

[B71] Siebert T. Rode C. Till O. Stutzig N. Blickhan R. (2016). Force reduction induced by unidirectional transversal muscle loading is independent of local pressure. J. Biomech. 49, 1156–1161. 10.1016/j.jbiomech.2016.02.053 26976226

[B72] Siebert T. Stutzig N. Rode C. (2018). A hill-type muscle model expansion accounting for effects of varying transverse muscle load. J. Biomech. 66, 57–62. 10.1016/j.jbiomech.2017.10.043 29154088

[B73] Siebert T. Till O. Blickhan R. (2014). Work partitioning of transversally loaded muscle: experimentation and simulation. Comput. Methods Biomech. Biomed. Eng. 17, 217–229. 10.1080/10255842.2012.675056 22515574

[B74] Simo J. C. Taylor R. L. (1991). Quasi-incompressible finite elasticity in principal stretches. continuum basis and numerical algorithms. Comput. Methods Appl. Mech. Eng. 85, 273–310. 10.1016/0045-7825(91)90100-K

[B75] Simo J. C. Taylor R. L. Pister K. S. (1985). Variational and projection methods for the volume constraint in finite deformation elasto-plasticity. Comput. Methods Appl. Mech. Eng. 51, 177–208. 10.1016/0045-7825(85)90033-7

[B76] Sjøgaard G. Savard G. Juel C. (1988). Muscle blood-flow during isometric activity and its relation to muscle fatigue. Eur. J. Appl. Physiol. Occup. Physiol. 57, 327–335. 10.1007/BF00635992 3371342

[B77] Sleboda D. A. Roberts T. J. (2017). Incompressible fluid plays a mechanical role in the development of passive muscle tension. Biol. Lett. 13:20160630. 10.1098/rsbl.2016.0630 28123108 PMC5310577

[B78] Smith L. R. Gerace-Fowler L. Lieber R. L. (2011). Muscle extracellular matrix applies a transverse stress on fibers with axial strain. J. Biomech. 44, 1618–1620. 10.1016/j.jbiomech.2011.03.009 21450292 PMC3085660

[B79] Tang C. Y. Tsui C. P. Stojanovic B. Kojic M. (2007). Finite element modelling of skeletal muscles coupled with fatigue. Int. J. Mech. Sci. 49, 1179–1191. 10.1016/j.ijmecsci.2007.02.002

[B80] Wakeling J. M. Blake O. M. Wong I. Rana M. Lee S. S. M. (2011). Movement mechanics as a determinate of muscle structure, recruitment and coordination. Philos. Trans. R. Soc. B Biol. Sci. 366, 1554–1564. 10.1098/rstb.2010.0294 21502126 PMC3130442

[B81] Wakeling J. M. Jackman M. Namburete A. I. (2013). The effect of external compression on the mechanics of muscle contraction. J. Appl. Biomech. 29, 360–364. 10.1123/jab.29.3.360 22927518

[B82] Wakeling J. M. Randhawa A. (2014). Transverse strains in muscle fascicles during voluntary contraction: a 2D frequency decomposition of B-mode ultrasound images. Int. J. Biomed. Imaging 4:352910 10.1155/2014/352910 PMC4195266 25328509

[B83] Weis-Fogh T. Alexander R. M. (1977). The sustained power output from straited muscle, in Scale Effects in Animal Locomotion, ed Pedley T. J. (New York: Academic Press), 511–525.

[B84] Weiss J. A. Maker B. N. Govindjee S. (1996). Finite element implementation of incompressible, transversely isotropic hyperelasticity. Comput. Method Appl. Mech. Eng. 135, 107–128. 10.1016/0045-7825(96)01035-3

[B85] Wickiewicz T. L. Roy R. R. Powell P. L. Edgerton V. R. (1983) Muscle architecture of the human lower limb. Clin. Orthop. Relat. Res. 179, 257–284. 10.1097/00003086-198310000-00042 6617027

[B86] Williams C. D. Regnier M. Daniel T. L. (2010). Axial and radial forces of cross-bridges depend on lattice spacing. PLoS Comput. Biol. 6:e1001018. 10.1371/journal.pcbi.1001018 21152002 PMC2996315

[B87] Williams C. D. Regnier M. Daniel T. L. (2012). Elastic energy storage and radial forces in the myofilament lattice depend on sarcomere length. PLoS Comput. Biol. 8:e1002770. 10.1371/journal.pcbi.1002770 23166482 PMC3499250

[B88] Winters T. M. Takahashi M. Lieber R. L. Ward S. R. (2011). Whole muscle length-tension relationships are accurately modeled as scaled sarcomeres in rabbit hindlimb muscles. J. Biomech. 44, 109–115. 10.1016/j.jbiomech.2010.08.033 20889156 PMC3003754

[B89] Woledge R. C. Curtin N. Homsher E. (1985). Energetic aspects of muscle contraction. Monogr. Physiol. Soc. 41, 1–357. 3843415

[B90] Wood L. K. Kayupov E. Gumucio J. P. Mendias C. L. Claflin D. R. Brooks S. V. (2014). Intrinsic stiffness of extracellular matrix increases with age in skeletal muscles of mice. J. Appl. Physiol. 117, 363–369. 10.1152/japplphysiol.00256.2014 24994884 PMC4137235

[B91] Yeh F. C. Verstynen T. D. Wang Y. B. Fernandez-Miranda J. C. Tseng W. Y. I. (2013). Deterministic diffusion fiber tracking improved by quantitative anisotropy. PLoS ONE 8:e80713. 10.1371/journal.pone.0080713 24348913 PMC3858183

[B92] Yeoh O. H. (1993). Some forms of the strain energy function for rubber. Rubber Chem. Tech. 66, 754–771. 10.5254/1.3538343

[B93] Yucesoy C. A. Huijing P. A. (2012). Specifically tailored use of the finite element method to study muscular mechanics within the context of fascial integrity: the linked fiber-matrix mesh model. Int. J. Multiscale Com. 10, 155–170. 10.1615/IntJMultCompEng.201100235612163314

[B94] Yucesoy C. A. Koopman B. H. F. J.M. Huijing P. A. Grootenboer H. J. (2002). Three-dimensional finite element modeling of skeletal muscle using a two-domain approach: linked fiber-matrix mesh model. J. Biomech. 35, 1253–1262. 10.1016/S0021-9290(02)00069-6 12163314

[B95] Zajac F. E. (1989). Muscle and tendon: properties, models, scaling, and application to biomechanics and motor control. Crit. Rev. Biomed. Eng. 17, 358–410. 2676342

[B96] Zuurbier C. J. Huijing P. A. (1993). Changes in muscle geometry of actively shortening unipennate rat gastrocnemius muscle. J. Morphol. 218, 167–180. 10.1002/jmor.1052180206 8263946

